# Biomimetic Entropy-Dominant Molecular Hinges with Picomolar Affinity

**DOI:** 10.1021/jacs.4c05274

**Published:** 2024-08-21

**Authors:** Zehuan Huang, Alexander S. Groombridge, Guanglu Wu, Magdalena Olesińska, Xiaoyi Chen, Jade A. McCune, Oren A. Scherman

**Affiliations:** Melville Laboratory for Polymer Synthesis, Yusuf Hamied Department of Chemistry, University of Cambridge, Lensfield Road, Cambridge CB2 1EW, United Kingdom

## Abstract

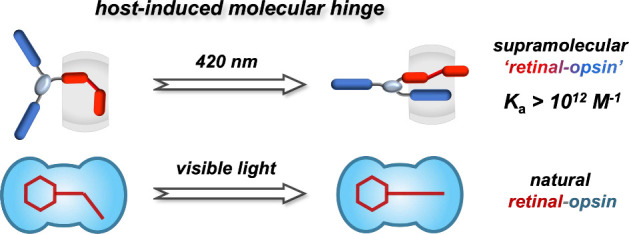

Molecular hinges are ubiquitous in both natural and artificial supramolecular systems. A major challenge to date, however, has been simultaneously achieving high thermodynamic and kinetic stability. Here, we employ host-enhanced intramolecular charge-transfer interactions to mediate entropy-favored complexation between a flexible AB_2_-type guest and a macrocyclic host, forming a new type of molecular hinge with an ultrahigh picomolar binding affinity (*K*_a_ > 10^12^ M^–1^). This entropy-promoted hinge modulates photoisomerization, exhibiting a substantial preference for the *E*-isomer, which is further demonstrated to mirror the natural retinal-opsin cycle, promoting the sensitization of visible light. This work unveils an efficient approach to exploit entropy-dominant architectures for the design of hierarchical molecular systems.

Molecular hinges are of great importance in natural^1−5^ and artificial^6−8^ self-assembled systems. Natural molecular hinges emerged from structural determination of human immunoglobulin G (1969),^1^ in which two heavy peptide chains are connected by interchain disulfide bridges. Several strategies have been exploited for the construction of artificial molecular hinges: directional folding through rotation of covalent bonds^7^ and intramolecular hinging through noncovalent interactions.^8^ Both suffer from a loss of conformational entropy, resulting in dynamic structures with limited thermodynamic stability.^9,10^ In natural molecular hinges, this entropy loss can be compensated by additional energy inputs (*e.g.*, ATP and light), resulting in an out-of-equilibrium state, which remains unexplored in artificial molecular hinges. Although significant advances have been made, designing ultrastable, entropy-favored molecular hinges remains a challenge.

On account of their high binding affinity, cucurbit[n]uril (CB[n])-mediated host–guest interactions have been extensively employed in supramolecular oligomers^11,12^ and polymers,^13−17^ dynamic hydrogels,^18^ molecular separators,^19^ protein/peptide assemblies,^20^ and other functional systems.^21,22^ CB[8] reinforces intermolecular π–π,^23^ polar−π,^24,25^ and charge-transfer^26^ interactions (Figure 1a). We envisaged designing intramolecular hinges through CB[8]-mediated complexation, whereby energy gained upon complexation compensates conformational entropic loss.

**Figure 1:**
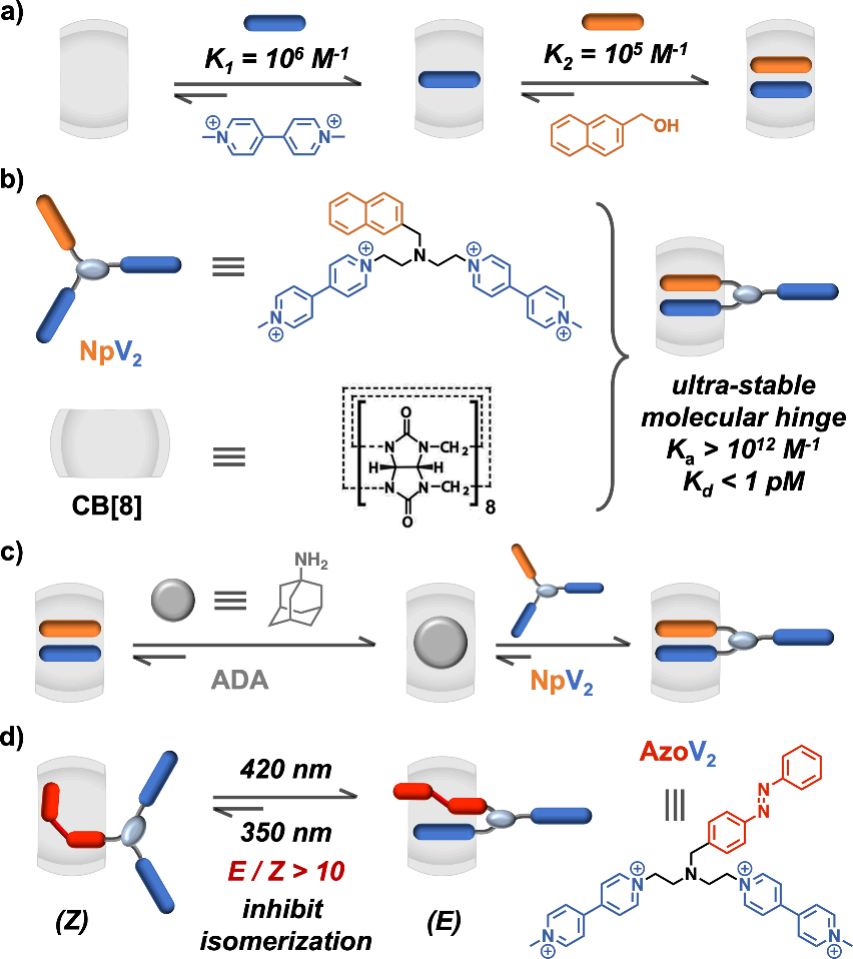
Schematics of (a) discrete CB[8]-mediated charge-transfer complexation between MV and Np; (b) molecular hinge formed from NpV_2_ and CB[8]; (c) sequential displacement; and (d) molecular structure of AzoV_2_ and control over its photoisomerization with CB[8]. Counterions are omitted for clarity.

We employ a macrocyclic host to enhance intramolecular charge-transfer interactions between donor and acceptor motifs (Figure 1b) within a tritopic AB_2_ guest. Compared to *inter*molecular complexation (Figure 1a), *intra*molecular complexation of adjacent donor–acceptor moieties circumvents translational and conformational entropic losses,^27−30^ offering a driving force for hinge formation. An extra donor–acceptor motif within the AB_2_ guest provides additional cooperativity, leading to substantial increases in binding affinity for molecular hinges.

As a proof of concept, an AB_2_ guest (NpV_2_), bearing two viologen derivatives (blue, electron acceptor) and one naphthyl moiety (orange, electron donor), was prepared (Figure 1b). An equimolar mixture of NpV_2_ and CB[8] resulted exclusively in a host-induced molecular hinge.^100^ Ultrahigh binding affinity was demonstrated through sequential competitive replacements, Figure 1c. Typically, 1-adamantylamine (ADA, 10^9^ M^–1^) readily displaces intermolecular donor–acceptor pairs from CB[8];^26^ here, the AB_2_ guest NpV_2_ replaced ADA, highlighting its ultrahigh affinity (10^12^ M^–1^). To demonstrate the use of external energy input for hinge stabilization, another AB_2_ guest (AzoV_2_) was prepared (Figure 1d). Complexation of AzoV_2_ with CB[8] enabled the fabrication of an artificial molecular hinge driven by light, mimicking light-assisted natural hinge systems.

Host-induced molecular hinge formation was studied through ^1^H NMR titrations, as shown in Figure 2a. Titration of free NpV_2_ (5.0 mM) into CB[8] (0.1 mM) at a molar ratio of 0.4 resulted in two sets of split peaks from two viologen environments, suggesting a 1:2 pseudostatic complex of NpV_2_-2CB[8] (Figure 2a). Continued titration to an equimolar ratio (1:1) led to a new series of sharp peaks. Compared to free NpV_2_, one set of the new viologen peaks (Ha–d) from NpV_2_-CB[8] exhibited a significant upfield shift, while the other displayed a considerable downfield shift (Ha′–d′). This arises from two distinct viologen environments (bound and unbound) within the 1:1 complex, confirming the formation of the NpV_2_-CB[8] molecular hinge.

**Figure 2:**
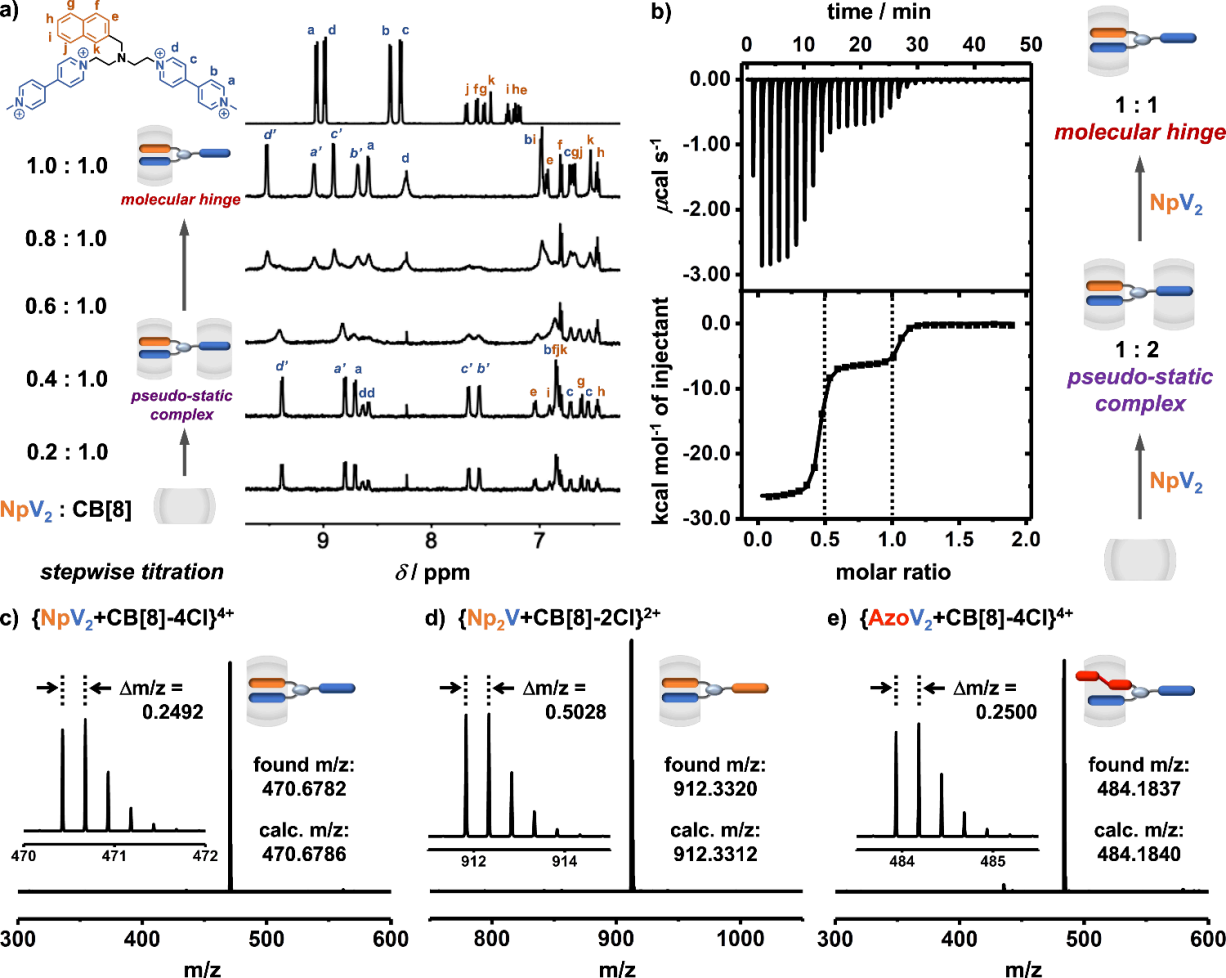
(a) ^1^H NMR spectra (D_2_O, 298 K) obtained through titration of NpV_2_ (5.0 mM) into CB[8] (0.1 mM), in which the proton peaks (a′, b′, c′, d′) refer to the viologen moiety locating outside the intramolecular charge-transfer complex; (b) ITC plot (H_2_O, 298 K) obtained through titration of NpV_2_ (1.0 mM) into CB[8] (0.1 mM); and (c) ESI-MS spectra for NpV_2_-CB[8] (1.0 mM), Np_2_V-CB[8] (1.0 mM), and AzoV_2_-CB[8] (1.0 mM). Counterions are omitted for clarity.

Isothermal titration calorimetry (ITC) confirmed stepwise binding; titration of NpV_2_ into CB[8] led to a binding curve with two clear transitions, Figure 2b. At a molar ratio of 0.5, a 1:2 complex (NpV_2_-2CB[8]) was formed in the presence of excess CB[8]. Further addition of NpV_2_ consumed the CB[8] from the weakly bound binary viologen (pseudostatic) complex, resulting in exclusive formation of the 1:1 NpV_2_-CB[8] species.

Generality of the AB_2_-CB[8] hinge design was further demonstrated with two additional AB_2_ guests (Np_2_V and AzoV_2_, Figures S2 and S3). Np_2_V inverted the number of viologen and naphthyl moieties, while AzoV_2_ replaced the naphthyl donor with a photoresponsive azobenzene. Overcoming typical ITC detection limits (10^3^ M^–1^ < *K*_a_ < 10^9^ M^–1^) was accomplished through competitive experiments with amantadine (ADA), indirectly yielding binding constants for the AB_2_-CB[8] hinges (see Figures S45 and S46); all thermodynamic parameters are summarized in Table 1. The AB_2_-CB[8] hinges exhibited *K*_a_ values over 10^12^ M^–1^, which unambiguously confirms a general ultrahigh affinity arising from this new design. These new molecular hinges were also observed in high-resolution ESI-MS with mass peaks (Figure 2c–e), consistent with their calculated *m*/*z* values.

**Table 1:** Thermodynamic Data for 1:1 Host–Guest Complexes with CB[8] Obtained through Sequential Competitive ITC

guest	*K*_a_ (M^–1^)	Δ*H* (kcal mol^–1^)	*T*Δ*S* (kcal mol^–1^)
NpV_2_	8.1 × 10^12^	–15.5 ± 0.2	2.1 ± 0.4
AzoV_2_	4.5 × 10^12^	–14.0 ± 0.1	3.3 ± 0.3
Np_2_V	1.0 × 10^12^	–14.7 ± 0.1	1.6 ± 0.3
ADA	3.1 × 10^9^	–8.2 ± 0.1	4.8 ± 0.2
MV	6.7 × 10^6^	–6.0 ± 0.1	3.3 ± 0.2

Formation of the pseudostatic 1:2 complex and the 1:1 molecular hinge was studied by diffusion-ordered NMR spectroscopy (DOSY). All diffusion coefficients (*D*) for the hinges exhibited similar values of 2.0 × 10^–10^ m^–2^ s^–1^ (Table 2), smaller than for free guests. This indicated an increase in the size of the molecular hinges compared to their free guest. Further reduction in *D* values was also observed for the 1:2 complexes of NpV_2_-2CB[8] and AzoV_2_-2CB[8] containing an additional CB[8]. These results are consistent with literature reports for discrete CB[8] complexes,^31^ rather than extended polymeric structures arising from intermolecular binding.^14−16^

**Table 2:** Diffusion Coefficients (*D*) for Free Guests and Their 1:1 and 1:2 Host–Guest Complexes with CB[8] Obtained by DOSY

		*D* (10^–10^ m^–2^ s^–1^)	
guest	free	+1.0 eq. CB[8]	+2.0 eq. CB[8]
NpV_2_	3.0 ± 0.5	2.0 ± 0.3	1.8 ± 0.3
AzoV_2_	2.8 ± 0.4	2.0 ± 0.4	1.7 ± 0.2
Np_2_V	3.1 ± 0.3	2.0 ± 0.3	-
CB[8]	2.9 ± 0.3	-	-

To quantify the role of entropy in the hinge formation, two sets of ITC experiments were performed. Titration of ADA into an intermolecular ternary complex of MV-CB[8]-Np resulted in an endothermic binding curve, Figure 3a. Despite its high enthalpic barrier, the translational entropic penalty of individual MV and Np guests bound within the 1:1:1 CB[8] complex was completely overcome by ADA displacement, resulting in an entropy-dominated process. Subsequent titration of NpV_2_ into ADA-CB[8] led to an exothermic binding curve, Figure 3b, indicating an enthalpic contribution toward hinge formation.

**Figure 3:**
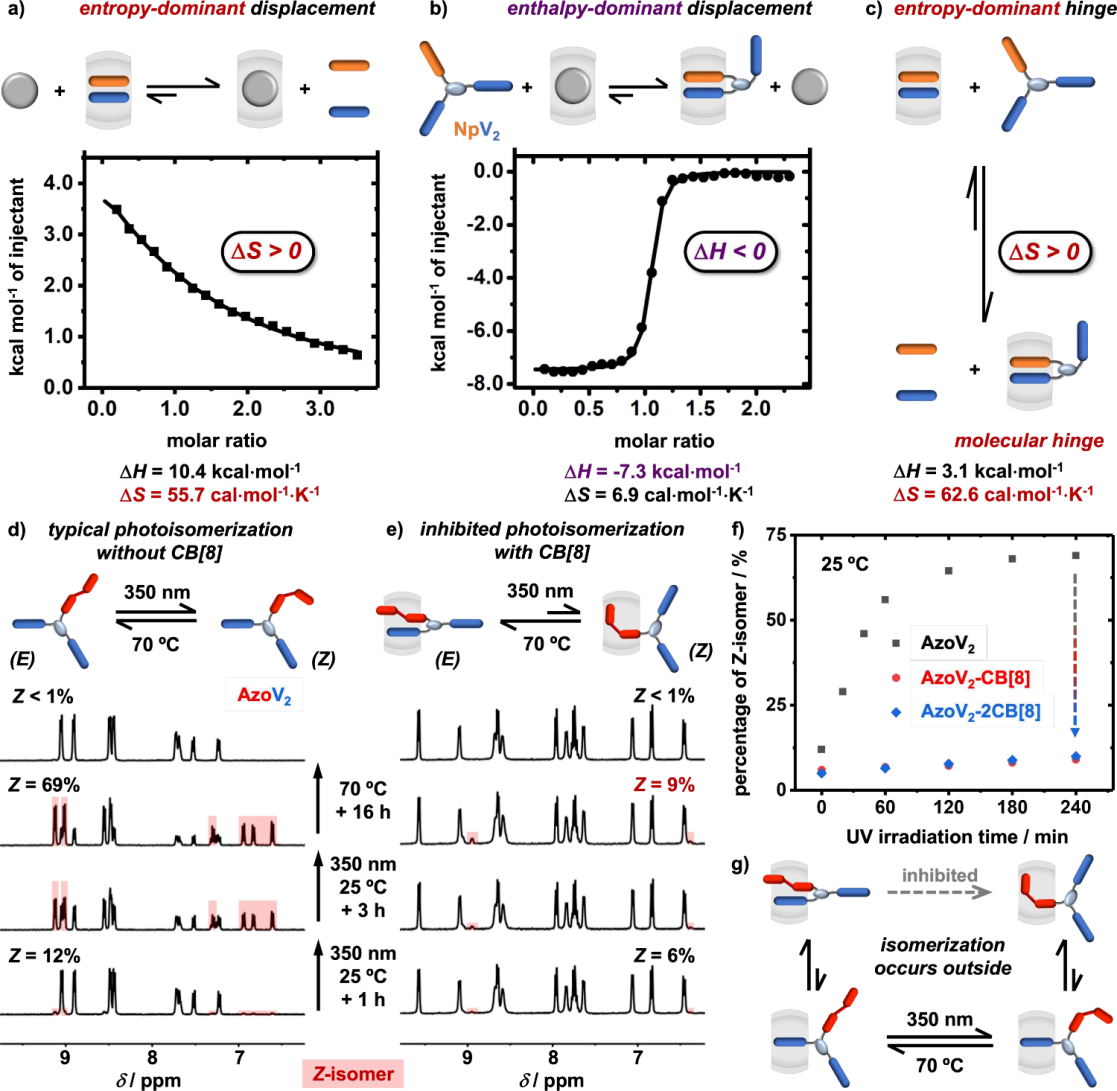
ITC titration plots (H_2_O, 298 K) of (a) ADA (0.5 mM) into MV-CB[8]-Np (0.03 mM) and (b) NpV_2_ (0.3 mM) into ADA-CB[8] (0.03 mM); (c) schematic equilibrium of competitive replacement of discrete MV and Np by NpV_2_ from CB[8]; ^1^H NMR spectra (D_2_O, 298 K) of (d) AzoV_2_ (1.0 mM) and (e) AzoV_2_-CB[8] (1.0 mM) obtained upon UV irradiation (λ = 350 nm) for 0, 1, and 4 h followed by subsequent heating at 70 °C for 16 h; (f) plot of percentage of *Z*-isomers in AzoV_2_, AzoV_2_-CB[8], and AzoV_2_-2CB[8]; (g) mechanism of inhibited photoisomerization.

Entropy and enthalpy changes for displacement of discrete MV and Np guests by NpV_2_ were determined through an equilibrium calculation based on both titrations. Formation of the AB_2_-CB[8] hinge is dominated by entropy with a Δ*S* value of 62.6 cal mol^–1^ K^–1^ (Figure 3c). This large entropy gain stems from the covalent linkage within NpV_2_, circumventing a translational entropic penalty. This confirms the importance of intramolecular interactions in the design and formation of stable AB_2_-CB[8] hinges. It is also worth noting that our AB_2_-type intramolecular hinge differs from previous reports on multifunctional enthalpy-driven systems (*e.g.*, A_3_ or A_3_ + B_2_) that preferentially form extended intermolecular structures^33,34^ as well as entropy-driven systems (*e.g.*, UPy-Napy) that favor formation of cyclic oligomers at low concentrations and linear polymers at high concentrations, limited by a concentration-dependent ring–chain equilibrium.^35,36^

To probe kinetic stability of AB_2_-CB[8] hinges, we employed AzoV_2_ and investigated its photoisomerization,^37−42^Figure 1d. Controllable photoisomerization of azobenzene within a discrete CB[8] ternary complex has been exploited in the fabrication of photoresponsive supramolecular systems.^37,39,41,42^ Introduction of an azobenzene-containing guest within the molecular hinge would favor the *E*-isomer and provide a definitive answer to the long-standing question of whether the *E* → *Z* photoisomerization takes place inside the CB[8] cavity or only occurs outside the cavity.

Photoisomerization of free AzoV_2_ and molecular hinges AzoV_2_-CB[8] and AzoV_2_-2CB[8] were monitored through time-dependent ^1^H NMR experiments after UV irradiation at 350 nm followed by heating at 70 °C. Free AzoV_2_ exhibits 12% *Z*-isomer at equilibrium (Figure 3d), which increased to 69% after 4 h UV irradiation. Subsequent heating led to quantitative transformation to the *E* isomer (*Z*-isomer <1%). Compared to free AzoV_2_, the percentage of the *Z*-isomer within AzoV_2_-CB[8] only displayed a slight increase from 6% at equilibrium to 9% following 4 h UV irradiation (350 nm), Figure 3e. This indicates a substantial preference for the *E*-isomer, on account of the pseudostatic AzoV_2_-CB[8] hinge that limits azobenzene dissociation from CB[8].

Quantitative results for *Z*/*E* photoisomerization were obtained from ^1^H NMR experiments (Figure 3f). Unlike free AzoV_2_ (black), formation of the *Z*-isomer was substantially suppressed in the complexed form of AzoV_2_–CB[8] (red) and AzoV_2_-2CB[8] (blue) after 4 h of UV irradiation. Therefore, *Z*/*E* photoisomerization is significantly inhibited inside a confined nanocavity (Figure 3g), answering the long-standing question: *E* → *Z* photoisomerization occurs solely in the free state outside the host cavity.

After elucidating the photoisomerization mechanism within the photoactive hinge, we employed AzoV_2_-CB[8] to devise a biomimetic supramolecular “retinal-opsin” cycle required for vision, Figure 4a.^43,44^ Natural retinal undergoes ATP-driven enzymatic chemoisomerization, enabling complexation with an opsin protein. This “retinal-opsin” complex is sensitive to visible light, allowing for photoisomerization back to the original retinal structure and subsequent release of retinal from the opsin protein, ready for the next cycle. Our synthetic system mirrors this cycle;^45^ both isomerization steps are driven by photoirradiation at different wavelengths, Figure 4b.

**Figure 4:**
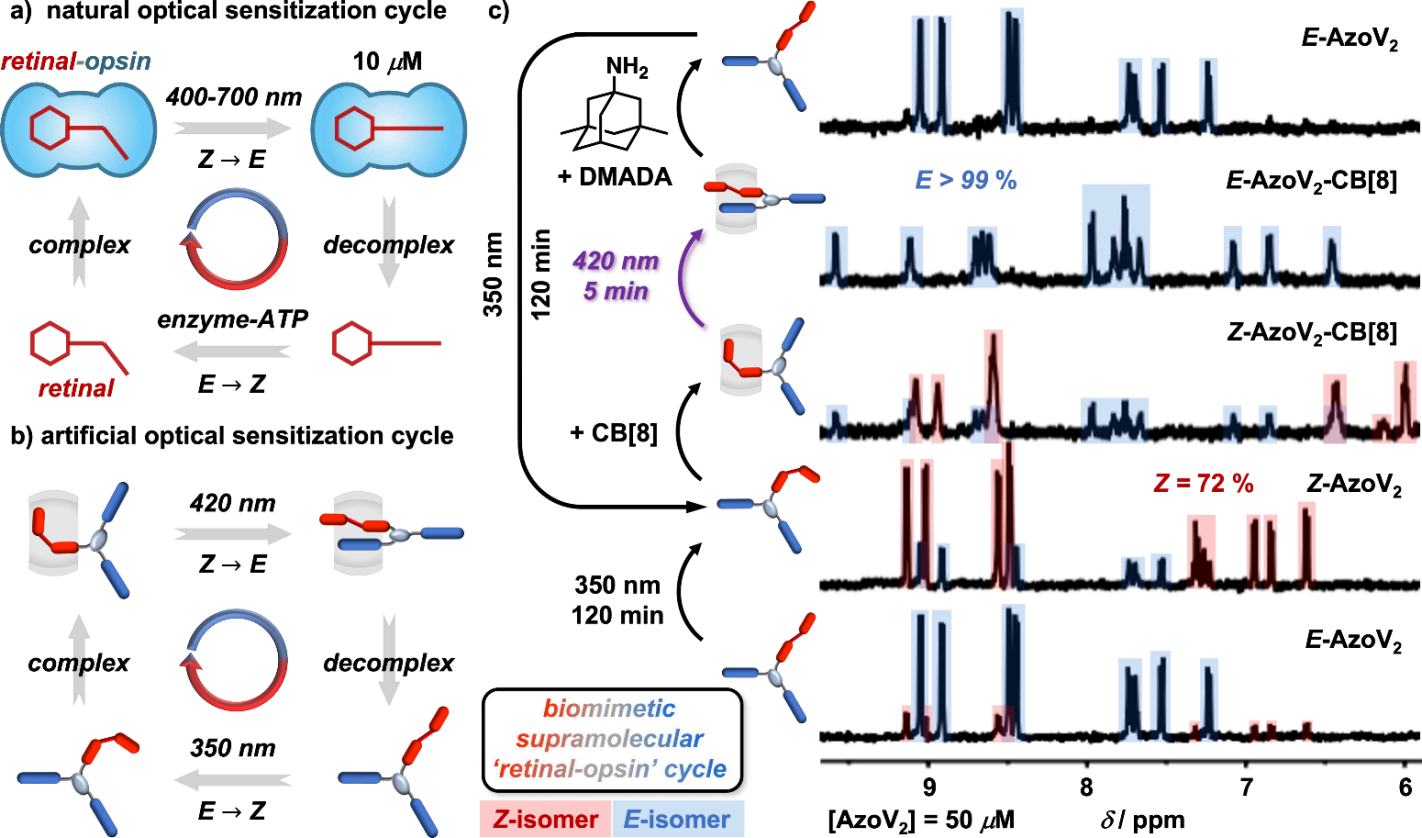
Schematics of (a) natural and (b) artificial optical sensitization cycle; (c) ^1^H NMR spectra (D_2_O, 298 K) of the biomimetic supramolecular “retinal-opsin” cycle. The concentration (50 μM) is comparable to that of retinal-opsin in the human eye,^32^ resulting in limited signal/noise ratio.

Figure 4c shows each step of the biomimetic cycle: (i) the AzoV_2_*E*-isomer undergoes photoisomerization at 350 nm to form the *Z*-isomer; (ii) CB[8] complexes with *Z*-AzoV_2_ through encapsulation of the azobenzene moiety; (iii) the *Z*-AzoV_2_-CB[8] complex undergoes photoisomerization with irradiation at 420 nm visible light to form the *E*-AzoV_2_-CB[8] complex (hinge); (iv) memantine(DMADA)-driven decomplexation releases *E*-AzoV_2_ from CB[8], ready for the next cycle. ^1^H NMR confirms quantitative formation of *E*-AzoV_2_-CB[8] upon photoirradiation at 420 nm after only 5 min (50 μM, comparable to the concentration of retinal-opsin found in the human eye^32^). Without CB[8] quantitative photoisomerization to *E*-AzoV_2_ does not occur (see Supporting Information Figure S48). Together with the data shown in Figure 3d and Figure 3e, these results show that the presence of CB[8], and formation of the AB_2_-CB[8] hinges, can significantly alter the ratio of *E*:*Z* isomers, allowing access to a quantitative (99%) solution of one isomer of azobenzene through noncovalent chemistries, without altering the chemical structure. This approach unveils a powerful route to selectively access isomers in ratios outside the photostationary state using reversible supramolecular chemistry.

Notably, the complexation of *trans* retinal with opsin is dominated by an entropic gain (Δ*S* = 34.6 cal mol^–1^ K^–1^, Δ*H* ≈ 0 kcal mol^–1^),^46^ similar to formation of the AB_2_-CB[8] hinge (Δ*S* = 62.6 cal mol^–1^ K^–1^, Δ*H* = 3.1 kcal mol^–1^), Figure 3c. Such a high affinity of the entropy-favored *E*-AzoV_2_-CB[8] complex leads to host-promoted isomerization, significantly lowering the overall free energy of the system. Both natural and artificial cycles contain four distinct steps, are sensitive to light and rely upon a single external chemical energy input (ATP & DMADA) ensuring cycle continuity. All the above highlights that AzoV_2_-CB[8] acts as a biomimetic supramolecular equivalent of the natural “retinal-opsin” complex, demonstrating an example of the artificial optical sensitization cycle.

In conclusion, we designed a new molecular hinge through host-enhanced charge-transfer interactions between a flexible, tritopic guest and a CB[8] macrocycle. Through entropy-dominant self-assembly, these molecular hinges possess picomolar binding with *K*_a_ > 10^12^ M^–1^, exhibiting ultrahigh thermodynamic affinity. Binding within the molecular hinge also displays high kinetic stability, inhibiting *E* → *Z* photoisomerization of azobenzene with a significant preference for the *E*-isomer and a relative ratio of *E*/*Z* over 1 order of magnitude. Furthermore, our supramolecular system driven by multistep processes mirrors that of the natural retinal-opsin cycle (4 steps, mediated by light, molecular recognition, and a single chemical input). This work introduces a general entropy-dominant approach to exploit ultrastable molecular hinges as a functional handle toward the design of molecular machines, artificial nanorobots, and biomimetic systems.

## Supporting Information Available

Instrumentation, synthesis of AB_2_-type guests, ^1^H and 2D NMR spectra for host–guest complexes, UV–vis spectra for photoisomerization, ITC titration plots and fitted curves (PDF)

## References

[ref1] Frangione B.; Milstein C.; Pink J. R. L. Immunoglobulins: Structural Studies of Immunoglobulin G. Nature 1969, 221, 145–148. 10.1038/221145a0. 5782707

[ref2] Sakano H.; Rogers J. H.; Hüppi K.; Brack C.; Traunecker A.; Maki R.; Wall R.; Tonegawa S. Domains and the hinge region of an immunoglobulin heavy chain are encoded in separate DNA segments. Nature 1979, 277, 627–633. 10.1038/277627a0. 106304

[ref3] Übelhart R.; Hug E.; Bach M. P.; Wossning T.; Dühren-von Minden M.; Horn A. H. C.; Tsiantoulas D.; Kometani K.; Kurosaki T.; Binder C. J.; Sticht H.; Nitschke L.; Reth M.; Jumaa H. Responsiveness of B cells is regulated by the hinge region of IgD. Nat. Immunol. 2015, 16, 534–543. 10.1038/ni.3141. 25848865

[ref4] Alt A.; Dang H. Q.; Wells O. S.; Polo L. M.; Smith M. A.; McGregor G. A.; Welte T.; Lehmann A. R.; Pearl L. H.; Murray J. M.; Oliver A. W. Specialized interfaces of Smc5/6 control hinge stability and DNA association. Nat. Commun. 2017, 8, 14011 10.1038/ncomms14011. 28134253 PMC5290277

[ref5] Zhou Y.; Yang L.; Duan J.; Cheng J.; Shen Y.; Wang X.; Han R.; Li H.; Li Z.; Wang L.; et al. Hinge region of Arabidopsis phyA plays an important role in regulating phyA function. Proc. Natl. Acad. Sci. U.S.A. 2018, 115, 11864–11873. 10.1073/pnas.1813162115. PMC6294956 30478060

[ref6] Feng L.; Wang Y.; Zhang K.; Wang K.-Y.; Fan W.; Wang X.; Powell J. A.; Guo B.; Dai F.; Zhang L.; Wang R.; Sun D.; Zhou H.-C. Molecular Pivot-Hinge Installation to Evolve Topology in Rare-Earth Metal–Organic Frameworks. Angew. Chem., Int. Ed. 2019, 58, 16682–16690. 10.1002/anie.201910717. 31518476

[ref7] Jones C. D.; Kershaw Cook L. J.; Marquez-Gamez D.; Luzyanin K. V.; Steed J. W.; Slater A. G. High-Yielding Flow Synthesis of a Macrocyclic Molecular Hinge. J. Am. Chem. Soc. 2021, 143, 7553–7565. 10.1021/jacs.1c02891. 33961419 PMC8397308

[ref8] Ai Y.; Chan M. H.-Y.; Chan A. K.-W.; Ng M.; Li Y.; Yam V. W.-W. A platinum(II) molecular hinge with motions visualized by phosphorescence changes. Proc. Natl. Acad. Sci. U.S.A. 2019, 116, 13856–13861. 10.1073/pnas.1908034116. 31243146 PMC6628644

[ref9] Tatko C. D.; Waters M. L. Selective Aromatic Interactions in beta-Hairpin Peptides. J. Am. Chem. Soc. 2002, 124, 9372–9373. 10.1021/ja0262481. 12167022

[ref10] Xiang J.; Jung J.-y.; Sampson N. S. Entropy Effects on Protein Hinges: The Reaction Catalyzed by Triosephosphate Isomerase. Biochemistry 2004, 43, 11436–11445. 10.1021/bi049208d. 15350130

[ref11] Wang J.; Huang Z.; Ma X.; Tian H. Visible-Light-Excited Room-Temperature Phosphorescence in Water by Cucurbit[8]uril-Mediated Supramolecular Assembly. Angew. Chem., Int. Ed. 2020, 59, 9928–9933. 10.1002/anie.201914513. 31799773

[ref12] Ma X.-K.; Zhang W.; Liu Z.; Zhang H.; Zhang B.; Liu Y. Supramolecular Pins with Ultralong Efficient Phosphorescence. Adv. Mater. 2021, 33, 2007476 10.1002/adma.202007476. 33660350

[ref13] Samanta S. K.; Quigley J.; Vinciguerra B.; Briken V.; Isaacs L. Cucurbit[7]uril Enables Multi-Stimuli-Responsive Release from the Self-Assembled Hydrophobic Phase of a Metal Organic Polyhedron. J. Am. Chem. Soc. 2017, 139, 9066–9074. 10.1021/jacs.7b05154. 28621947 PMC5570531

[ref14] Huang Z.; Yang L.; Liu Y.; Wang Z.; Scherman O. A.; Zhang X. Supramolecular Polymerization Promoted and Controlled through Self-Sorting. Angew. Chem., Int. Ed. 2014, 53, 5351–5355. 10.1002/anie.201402817. 24711345

[ref15] Raeisi M.; Kotturi K.; del Valle I.; Schulz J.; Dornblut P.; Masson E. Sequence-Specific Self-Assembly of Positive and Negative Monomers with Cucurbit[8]uril Linkers. J. Am. Chem. Soc. 2018, 140, 3371–3377. 10.1021/jacs.7b13304. 29444409

[ref16] Yin Z.; Song G.; Jiao Y.; Zheng P.; Xu J.-F.; Zhang X. Dissipative Supramolecular Polymerization Powered by Light. CCS Chem. 2019, 1, 335–342. 10.31635/ccschem.019.20190013.

[ref17] Yang B.; Zhang X.-D.; Li J.; Tian J.; Wu Y.-P.; Yu F.-X.; Wang R.; Wang H.; Zhang D.-W.; Liu Y.; Zhou L.; Li Z.-T. In Situ Loading and Delivery of Short Single- and Double-Stranded DNA by Supramolecular Organic Frameworks. CCS Chem. 2019, 1, 156–165. 10.31635/ccschem.019.20180011.

[ref18] Appel E. A.; Biedermann F.; Rauwald U.; Jones S. T.; Zayed J. M.; Scherman O. A. Supramolecular Cross-Linked Networks via Host–Guest Complexation with Cucurbit[8]uril. J. Am. Chem. Soc. 2010, 132, 14251–14260. 10.1021/ja106362w. 20845973

[ref19] Wu H.; Wang Y.; Jones L. O.; Liu W.; Zhang L.; Song B.; Chen X.-Y.; Stern C. L.; Schatz G. C.; Stoddart J. F. Selective Separation of Hexachloroplatinate(IV) Dianions Based on Exo-Binding with Cucurbit[6]uril. Angew. Chem., Int. Ed. 2021, 60, 17587–17594. 10.1002/anie.202104646. 34031957

[ref20] Hirani Z.; Taylor H. F.; Babcock E. F.; Bockus A. T.; Varnado C. D. Jr; Bielawski C. W.; Urbach A. R. Molecular recognition of methionine-terminated peptides by cucurbit [8] uril. J. Am. Chem. Soc. 2018, 140, 12263–12269. 10.1021/jacs.8b07865. 30221936 PMC6312855

[ref21] Jang Y.; Jang M.; Kim H.; Lee S. J.; Jin E.; Koo J. Y.; Hwang I.-C.; Kim Y.; Ko Y. H.; Hwang I.; Oh J. H.; Kim K. Point-of-Use Detection of Amphetamine-Type Stimulants with Host-Molecule-Functionalized Organic Transistors. Chem. 2017, 3, 641–651. 10.1016/j.chempr.2017.08.015.

[ref22] He S.; Biedermann F.; Vankova N.; Zhechkov L.; Heine T.; Hoffman R. E.; De Simone A.; Duignan T. T.; Nau W. M. Cavitation energies can outperform dispersion interactions. Nat. Chem. 2018, 10, 1252–1257. 10.1038/s41557-018-0146-0. 30297753

[ref23] Jiao D.; Biedermann F.; Tian F.; Scherman O. A. A systems approach to controlling supramolecular architecture and emergent solution properties via host- guest complexation in water. J. Am. Chem. Soc. 2010, 132, 15734–15743. 10.1021/ja106716j. 20945904

[ref24] Huang Z.; Chen X.; Wu G.; Metrangolo P.; Whitaker D.; McCune J. A.; Scherman O. A. Host-Enhanced Phenyl-Perfluorophenyl Polar-π Interactions. J. Am. Chem. Soc. 2020, 142, 7356–7361. 10.1021/jacs.0c02275. 32248683 PMC7181256

[ref25] Huang Z.; Chen X.; O’Neill S.; Wu G.; Whitaker D. J.; Li J.; McCune J. A.; Scherman O. A. Highly Compressible Glass-like Supramolecular Polymer Networks. Nat. Mater. 2022, 21, 103–109. 10.1038/s41563-021-01124-x. 34819661

[ref26] Kim H.-J.; Heo J.; Jeon W. S.; Lee E.; Kim J.; Sakamoto S.; Yamaguchi K.; Kim K. Selective Inclusion of a Hetero-Guest Pair in a Molecular Host: Formation of Stable Charge-Transfer Complexes in Cucurbit[8]uril. Angew. Chem., Int. Ed. 2001, 40, 1526–1529. 10.1002/1521-3773(20010417)40:8<1526::AID-ANIE1526>3.0.CO;2-T. 29712342

[ref27] Page M. I.; Jencks W. P. Entropic Contributions to Rate Accelerations in Enzymic and Intramolecular Reactions and the Chelate Effect. Proc. Natl. Acad. Sci. U.S.A. 1971, 68, 1678–1683. 10.1073/pnas.68.8.1678. 5288752 PMC389269

[ref28] Page M. I. Entropy, Binding Energy, and Enzymic Catalysis. Angew. Chem., Int. Ed. 1977, 16, 449–459. 10.1002/anie.197704491.

[ref29] Menger F. M. On the source of intramolecular and enzymatic reactivity. Acc. Chem. Res. 1985, 18, 128–134. 10.1021/ar00113a001.

[ref30] Smith G. M.; Carpenter J. D.; Marks T. J. Intramolecular vs. intermolecular alkyl carbon-hydrogen bond activation. Complete thermodynamic and kinetic parameters for a reversible cyclometalation. J. Am. Chem. Soc. 1986, 108, 6805–6807. 10.1021/ja00281a059.

[ref100] Groombridge A. S. Aqueous Self-Assembly with Cucurbit[n]urils: From Solution to Emulsion, Ph.D. Thesis, University of Cambridge, 2017.

[ref31] Clarke D. E.; Wu G.; Wu C.; Scherman O. A. Host–Guest Induced Peptide Folding with Sequence-Specific Structural Chirality. J. Am. Chem. Soc. 2021, 143, 6323–6327. 10.1021/jacs.1c00342. 33860670 PMC8154536

[ref32] Bridges C. D.; Alvarez R. A.; Fong S. L. Vitamin A in human eyes: amount, distribution, and composition. Investig. Ophthalmol. Vis. Sci. 1982, 22, 706–714. 7076416

[ref33] Zhang K.-D.; Tian J.; Hanifi D.; Zhang Y.; Sue A. C.-H.; Zhou T.-Y.; Zhang L.; Zhao X.; Liu Y.; Li Z.-T. Toward a Single-Layer Two-Dimensional Honeycomb Supramolecular Organic Framework in Water. J. Am. Chem. Soc. 2013, 135, 17913–17918. 10.1021/ja4086935. 24079461

[ref34] Pfeffermann M.; Dong R.; Graf R.; Zajaczkowski W.; Gorelik T.; Pisula W.; Narita A.; Müllen K.; Feng X. Free-Standing Monolayer Two-Dimensional Supramolecular Organic Framework with Good Internal Order. J. Am. Chem. Soc. 2015, 137, 14525–14532. 10.1021/jacs.5b09638. 26529142 PMC4749122

[ref35] Scherman O. A.; Ligthart G. B. W. L.; Sijbesma R. P.; Meijer E. W. A Selectivity-Driven Supramolecular Polymerization of an AB Monomer. Angew. Chem., Int. Ed. 2006, 45, 2072–2076. 10.1002/anie.200504192. 16502447

[ref36] de Greef T. F. A.; Ercolani G.; Ligthart G. B. W. L.; Meijer E. W.; Sijbesma R. P. Influence of Selectivity on the Supramolecular Polymerization of AB-Type Polymers Capable of Both A· A and A· B Interactions. J. Am. Chem. Soc. 2008, 130, 13755–13764. 10.1021/ja8046409. 18800796

[ref37] Tian F.; Jiao D.; Biedermann F.; Scherman O. A. Orthogonal switching of a single supramolecular complex. Nat. Commun. 2012, 3, 1207 10.1038/ncomms2198. 23149751

[ref38] del Barrio J.; Horton P. N.; Lairez D.; Lloyd G. O.; Toprakcioglu C.; Scherman O. A. Photocontrol over Cucurbit[8]uril Complexes: Stoichiometry and Supramolecular Polymers. J. Am. Chem. Soc. 2013, 135, 11760–11763. 10.1021/ja406556h. 23879174

[ref39] Stoffelen C.; Voskuhl J.; Jonkheijm P.; Huskens J. Dual Stimuli-Responsive Self-Assembled Supramolecular Nanoparticles. Angew. Chem., Int. Ed. 2014, 53, 3400–3404. 10.1002/anie.201310829. 24615852

[ref40] del Barrio J.; Ryan S. T. J.; Jambrina P. G.; Rosta E.; Scherman O. A. Light-Regulated Molecular Trafficking in a Synthetic Water-Soluble Host. J. Am. Chem. Soc. 2016, 138, 5745–5748. 10.1021/jacs.5b11642. 26876686

[ref41] Gao C.; Huang Q.; Lan Q.; Feng Y.; Tang F.; Hoi M. P. M.; Zhang J.; Lee S. M. Y.; Wang R. A user-friendly herbicide derived from photo-responsive supramolecular vesicles. Nat. Commun. 2018, 9, 2967 10.1038/s41467-018-05437-5. 30054483 PMC6063903

[ref42] Fregoni J.; Granucci G.; Persico M.; Corni S. Strong Coupling with Light Enhances the Photoisomerization Quantum Yield of Azobenzene. Chem. 2020, 6, 250–265. 10.1016/j.chempr.2019.11.001.

[ref43] Wald G. Vitamin A in the Retina. Nature 1933, 132, 316–317. 10.1038/132316a0.

[ref44] Wald G. Carotenoids and the Vitamin A Cycle in Vision. Nature 1934, 134, 65–65. 10.1038/134065a0.

[ref45] Wald G. Molecular Basis of Visual Excitation. Science 1968, 162, 230–239. 10.1126/science.162.3850.230. 4877437

[ref46] Noy N.; Xu Z. J. Thermodynamic parameters of the binding of retinol to binding proteins and to membranes. Biochemistry 1990, 29, 3888–3892. 10.1021/bi00468a014. 2354160

