# Supplementary material for: Biomimetic Entropy-Dominant Molecular Hinges with Picomolar Affinity
Source: J Am Chem Soc. 2024 Aug 21;146(35):24244–9. doi: 10.1021/jacs.4c05274 (PMC11378272; doi:10.1021/jacs.4c05274)
Supplement: Supplementary file 1 — ja4c05274_si_001.pdf [file ja4c05274_si_001.pdf]

Biomimetic Entropy-Dominant Molecular Hinges with Picomolar Affinity

Zehuan Huang[†], Alexander S. Groombridge[†], Guanglu Wu, Magdalena Olesińska, Xiaoyi Chen, Jade A. McCune, Oren A. Scherman*

Melville Laboratory for Polymer Synthesis, Yusuf Hamied Department of Chemistry, University of Cambridge, Cambridge, CB2 1EW, UK.
** Corresponding author, E-mail: oas23@cam.ac.uk*
[†] These authors contributed equally to this work.

Contents

1 Supplementary Information **3**
- 1.1 Experimental . . . 3
- 1.2 Instrumentation . . . 3
- 1.3 NpV_2 Synthesis . . . 4
  - 1.3.1 1-methyl-[4,4'-bipyridin]-1-ium iodide; Me-Bipy . . . 4
  - 1.3.2 2,2'-((naphthalen-2-ylmethyl)azanediyl)bis(ethan-1-ol) 4
  - 1.3.3 2-bromo-*N*-(2-bromoethyl)-*N*-(naphthalen-2-ylmethyl)-ethan-1-amine . . . 5
  - 1.3.4 1',1"'-(((naphthalen-2-ylmethyl)azanediyl)bis(ethane-2,1-diyl))bis(1-methyl[4,4'-bipyridine]-1,1'-diium) tetrachloride; NpV_2 . . . 5
- 1.4 $AzoV_2$ Synthesis . . . 7
  - 1.4.1 (4-(phenyldiazenyl)phenyl)methanol . . . 7
  - 1.4.2 2,2'-((4-(phenyldiazenyl)benzyl)azanediyl)bis(ethan-1-ol) . . . 7
  - 1.4.3 2-bromo-*N*-(2-bromoethyl)-*N*-(4-(phenyldiazenyl)benzyl)-ethan-1-amine . . . 8
  - 1.4.4 1',1"'-(((4-(phenyldiazenyl)benzyl)azanediyl)bis(ethane-2,1-diyl))bis(1-methyl-[4,4'-bipyridine]-1,1'-diium) tetrachloride; $AzoV_2$ . . . 9
- 1.5 Np_2V Synthesis . . . 10
  - 1.5.1 2-(bis(naphthalen-2-ylmethyl)amino)ethan-1-ol . . . 10
  - 1.5.2 2-bromo-*N*,*N*-bis(naphthalen-2-ylmethyl)ethan-1-amine 11
  - 1.5.3 1-(2-(bis(naphthalen-2-ylmethyl)amino)ethyl)-1'-methyl-[4,4'-bipyridine]-1,1'-diium tetrachloride; Np_2V . . . 11

1.6 2D NMR . . . 13
1.6.1 NpV_2 . . . 14
1.6.2 $AzoV_2$ . . . 21
1.6.3 Np_2V . . . 27
1.7 ^1H NMR Titrations . . . 29
1.7.1 NpV_2-CB[8] . . . 29
1.7.2 NpV_2-CB[7] . . . 29
1.7.3 $AzoV_2$-CB[8] . . . 32
1.7.4 $AzoV_2$-CB[7] . . . 34
1.7.5 $AzoV_2$-CB[8]-CB[7] . . . 36
1.7.6 Np_2V-CB[8] . . . 37
1.7.7 Np_2Vio-CB[7] . . . 38
1.8 Photo-isomerisation of $AzoV_2$ Complexes . . . 41
1.9 Further Complexation with NpV_2-2CB[8] . . . 48
1.10 Mixtures with ADA·HCl . . . 50
1.11 UV-Vis Spectroscopy . . . 53
1.12 ITC . . . 55

1 Supplementary Information

1.1 Experimental

1.2 Instrumentation

All chemicals were purchased from Sigma Aldrich at the highest purity available and used as received unless otherwise specified.

^{1}H, ^{13}C and DOSY NMR spectra were recorded using a Bruker Avance 400 QNP, a Bruker 500 DCH Cryoprobe, and a Bruker Avance 500 III HD Smart Probe. Chemical Shifts are recorded in ppm in $CDCl_3$, D_2O and d_6-DMSO with internal references set to δ 7.26 ppm, 4.79 ppm, and 2.50 ppm respectively. 2D COSY, HSQC, HMBC and DOSY were carried out where appropriate. ATR FT-IR spectroscopy was performed using a PerkinElmer Spectrum 100 series FT-IR spectrometer equipped with a universal ATR sampling accessory. UV-Vis spectra were recorded on a Varian Cary 4000 UV-Vis spectrophotometer in aqueous solutions with 1 nm resolution at 25 °C. Photoirradiation was carried out using a LZC-ORG photoreactor from Luzchem Research Inc. equipped with UVA lamps centred at 360 nm. ITC experiments were carried out on a Malvern MicroCal ITC200 at 298.15 K in MilliQ water. Raw data was processed and integrated with NITPIC (v1.2.0), fitted in Sedphat (v12.1b), and visualised through GUSSI (v1.1.0). Error estimations were carried out by F statistics at the 0.68 confidence level.[1,2]

1.3 NpV_2 Synthesis

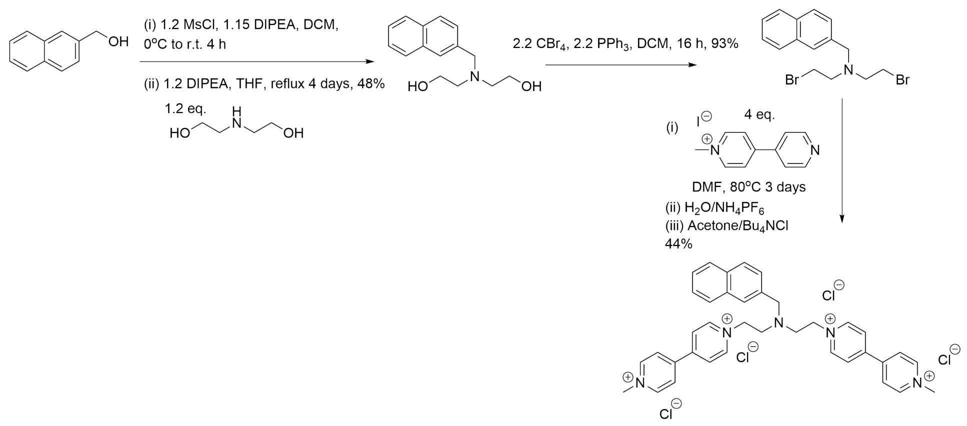

Figure 1: Scheme of NpV_2 synthesis.

1.3.1 1-methyl-[4,4'-bipyridin]-1-ium iodide; Me-Bipy

The synthesis was adapted from the literature.[3] 4,4'-bipyridine (24.7 g, 0.16 mol) was dissolved in DCM (500 mL). Iodomethane (28.37 g, 0.20 mol) was added in one portion, and the reaction was left for 16 h. The product precipitant was filtered and washed with further DCM (100 mL) to give the product as a yellow solid (37.10 g, 79 %). FT-IR ν_{max}/cm^{-1} 3078w, 3020br, 1648s, 1639s, 1602, 1579w, 1547s, 1524, 1498, 1467w, 1416s. δH (D_2O, 400 MHz) 8.91 (2H, d, 6.54 Hz), 8.78 (2H, d, 6.17 Hz), 8.39 (2H, d, 6.43 Hz), 7.92 (2H, d, 6.20 Hz), 4.45 (3H, s) ppm. δC (D_2O, 100 MHz) 153.5 (ArC), 149.9 (ArCH), 145.6 (ArCH), 142.6 (ArC), 125.8 (ArCH), 122.5 (ArCH), 47.8 (CH_3) ppm.

1.3.2 2,2'-((naphthalen-2-ylmethyl)azanediyl)bis(ethan-1-ol)

2-naphthalenemethanol (1.75 g, 11.1 mmol) was dissolved in anhydrous DCM (35 mL) under N_2 and cooled to 0 °C by an ice bath. Methanesulfonyl chloride (1.03 mL, 13.3 mmol) was added, followed by dropwise addition of *N*,*N*-diisopropylethylamine (3.30 mL, 12.7 mmol). The ice bath was removed, and the reaction stirred for 4 h. After dilution with DCM (10 mL), the mixture was washed with saturated NH_4Cl solution twice, dried with anhydrous $MgSO_4$, and the solvent was removed under reduced pressure to give the crude activated alcohol. Diethanolamine (1.42 g, 13.5 mmol)

was dissolved in anhydrous THF (25 mL) under N_2. The crude activated alcohol was dissolved in anhydrous THF (5 mL), and was added by syringe to the amine followed by dropwise addition of *N*,*N*-diisopropylethylamine (3.40 mL, 13.1 mmol). The mixture was refluxed at 70 °C for 3 days. Solvent was removed under reduced pressure and purification was carried out by SiO_2 column chromatography in a mixture of DCM:MeOH 95:5 to give the product as a brown oil (1.30 g, 48 %). TLC (SiO_2) R_f = 0.26 DCM:MeOH 95:5. FT-IR ν_{max}/cm^{-1} 3342br, 3053, 2948br, 2880br, 2819br, 1634w, 1602s, 1509s, 1449br. δH (d_6-DMSO, 400 MHz) 7.84-7.90 (3H, m), 7.81 (1H, s), 7.54 (1H, dd, 8.42, 1.51 Hz), 7.45-7.52 (2H, m), 4.38 (2H, t, 5.43 Hz), 3.80 (2H, s), 3.49 (4H, q, 6.03 Hz), 2.60 (4H, t, 6.36 Hz) ppm. δC (d_6-DMSO, 100 MHz) 137.7 (ArC), 132.9 (ArC), 132.3 (ArC), 127.5 (ArCH, 3 overlapped), 127.3 (ArCH), 126.7 (ArCH), 125.9 (ArCH), 125.5 (ArCH), 59.2 (CH_2, 2 overlapped), 56.4 (CH_2) ppm. HRMS calculated for $[M + H]^+$: $C_{15}H_{20}NO_2^+$ 246.1491, found 246.1491.

1.3.3 2-bromo-*N*-(2-bromoethyl)-*N*-(naphthalen-2-ylmethyl)ethan-1-amine

2,2'-((naphthalen-2-ylmethyl)azanediyl)bis(ethan-1-ol) (911 mg, 3.71 mmol) was dissolved in anhydrous DCM (37 mL) and tetrabromomethane (2.71 g, 8.18 mmol) was added, followed by triphenylphosphine (2.14 g, 8.16 mmol). The reaction was stirred at ambient conditions under N_2 for 16 h. The reaction mixture was washed with aqueous *sat.* $NaHCO_3$ x2 and brine x1. The organic phase was dried with anhydrous $MgSO_4$ before removal of solvent by rotary evaporation. Purification was carried out by SiO_2 column chromatography in a mixture of petroleum ether 40-60 and ethyl acetate 6:1 to give the product as a brown oil that crystallised upon standing (1.28 g, 93 %). TLC (SiO_2) R_f = 0.71 Pet:EtOAc 6:1. δH ($CDCl_3$, 400 MHz) 7.79-7.86 (3H, m), 7.75 (1H, s), 7.44-7.54 (3H, m), 3.89 (2H, s), 3.38 (4H, t, 7.25 Hz), 3.03 (4H, t, 7.24 Hz) ppm. δC ($CDCl_3$, 100 MHz) 136.5 (ArC), 133.4 (ArC), 133.2 (ArC), 128.4 (ArCH), 127.9 (ArCH), 127.3 (ArCH), 126.9 (ArCH), 126.3 (ArCH), 125.9 (ArCH), 59.2 (CH_2), 56.4 (CH_2), 30.1 (CH_2) ppm. HRMS calculated for $[M + H]^+$: $C_{15}H_{18}NBr_2^+$ 369.9806, found 369.9812.

1.3.4 1',1"'-(((naphthalen-2-ylmethyl)azanediyl)bis(ethane-2,1-diyl))bis(1-methyl[4,4'-bipyridine]-1,1'-diium) tetrachloride; NpV_2

1-methyl-[4,4'-bipyridin]-1-ium iodide (4.14 g, 13.89 mmol) was dissolved in anhydrous DMF (40 mL) under N_2 at 80 °C. 2-bromo-*N*-(2-bromoethyl)-

N-(naphthalen-2-ylmethyl)ethan-1-amine (1.28 g, 3.47 mmol) in anhydrous DMF (4 mL) was added in one portion, and the mixture stirred for 3 days at 80 °C. The product precipitant was filtered and washed with further DMF (20 mL) and ACN (40 mL) to give a brown amorphous solid (1.65 g). This mixed iodide/bromide salt was dissolved in the minimum volume of water, to which a saturated aqueous solution of ammonium hexafluorophosphate was added until no further precipitation occurred. The product was separated by filtration and dried. The product as a hexafluorophosphate salt was then dissolved in acetone, to which a saturated acetone solution of tetrabutylammonium chloride was added until no further precipitation occurred. The product was filtered off and dried to give a brown amorphous solid (1.05 g, 44 %). FT-IR ν_{max}/cm^{-1} 3364br, 3107w, 3028br, 2853w, 1637s, 1559, 1508, 1442. λ_{max}(H_2O)/nm 223 and 260 (ϵ/dm^3 mol^{-1} cm^{-1} 69844 and 41325). δH (D_2O, 500 MHz) 9.06 (4H, d, 6.74 Hz), 8.99 (4H, d, 6.90 Hz), 8.38 (4H, d, 6.85 Hz), 8.28 (4H, d, 6.86 Hz), 7.68 (1H, d, 8.11 Hz), 7.58 (1H, d, 8.38 Hz), 7.51 (1H, d, 8.11 Hz), 7.45 (1H, s), 7.29 (1H, t, 7.51 Hz), 7.23 (1H, t, 7.50 Hz), 7.19 (1H, dd, 8.39, 1.50 Hz), 4.91 (4H, t, 6.31 Hz), 4.54 (3H, s), 3.82 (2H, s), 3.58 (4H, t, 6.33 Hz) ppm. δC (D_2O, 125 MHz) 149.2 (VC), 148.7 (VC), 146.2 (VCH), 145.4 (VCH), 136.3 (NpC), 132.8 (NpC), 131.8 (NpC), 128.1 (NpCH), 128.0 (NpCH, 2 overlapped), 127.5 (NpCH), 127.4 (NpCH), 126.7 (NpCH), 126.4 (VCH), 126.2 (VCH, NpCH overlapped), 59.6 (CH_2), 58.0 (CH_2), 54.1 (CH_2), 48.3 (CH_3) ppm. HRMS calculated for $[M$ - Cl$]^+$: $C_{37}H_{39}N_5{}^{35}Cl_3{}^+$ 658.2266, found 658.2236.

1.4 AzoV$_2$ Synthesis

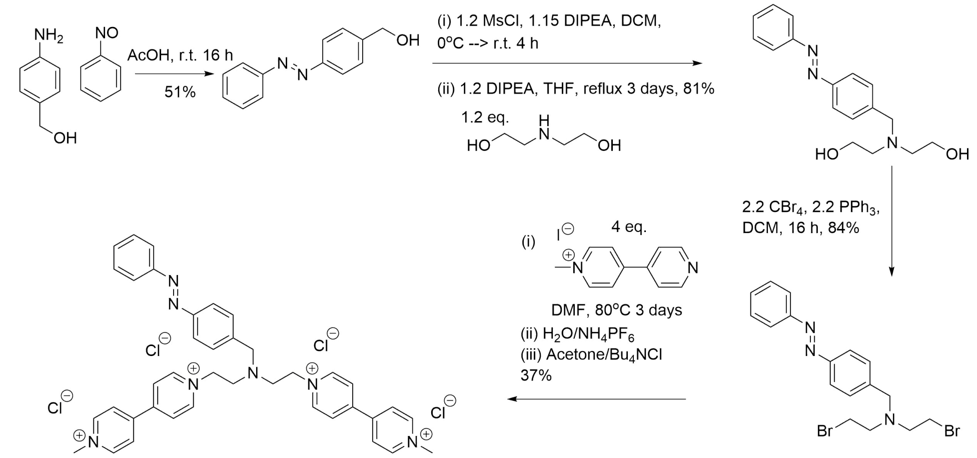

Figure 2: Scheme of AzoV$_2$ synthesis.

1.4.1 (4-(phenyldiazenyl)phenyl)methanol

The synthesis was carried out according to literature procedures.[4] The product was obtained as an orange crystalline solid (4.38 g, 51 %). TLC (SiO_2) $R_f = 0.30$ Pet:EtOAc 3:1. FT-IR ν_{max}/cm^{-1} 3310br, 3059w, 2915w, 2863w, 1659w, 1584, 1527w, 1504w, 1487w, 1462w, 1442. δH ($CDCl_3$, 400 MHz) 7.90-7.94 (4H, m), 7.45-7.55 (5H, m), 4.79 (2H, s) ppm. δC ($CDCl_3$, 100 MHz) 152.8 (ArC), 152.3 (ArC), 144.0 (ArC), 131.1 (ArCH), 129.2 (ArCH), 127.6 (ArCH), 123.2 (ArCH), 123.0 (ArCH), 65.0 (CH_2) ppm.

1.4.2 2,2'-((4-(phenyldiazenyl)benzyl)azanediyl)bis(ethan-1-ol)

The synthesis was carried out similarly to that in section 1.3.2. (4-(phenyldiazenyl)phenyl)methanol (1.50 g, 7.08 mmol) was dissolved in anhydrous DCM (50 mL) under N_2 and cooled to 0 °C by an ice bath. Methanesulfonyl chloride (0.66 mL, 8.53 mmol) was added, followed by dropwise addition of *N*,*N*-diisopropylethylamine (2.15 mL, 8.29 mmol). The ice bath was removed, and the reaction stirred for 4 h. After dilution with DCM (10 mL), the mixture was washed with saturated NH_4Cl solution twice, dried with anhydrous $MgSO_4$, and the solvent was removed under reduced pressure to give the crude activated alcohol. Diethanolamine (897 mg, 8.53 mmol) was

dissolved in anhydrous THF (12 mL) under N_2. The crude activated alcohol was dissolved in anhydrous THF (12 mL), and was added by syringe to the amine followed by dropwise addition of *N*,*N*-diisopropylethylamine (2.20 mL, 8.48 mmol). The mixture was refluxed at 70 °C for 3 days. Solvent was removed under reduced pressure and purification was carried out by SiO_2 column chromatography in a mixture of DCM:MeOH 95:5 to give the product as an orange oil that crystallised upon standing (1.72 g, 81 %). TLC (SiO_2) R_f = 0.33 DCM:MeOH 95:5. FT-IR ν_{max}/cm^{-1} 3293br, 3200br, 2948s, 2871br, 2836w, 1656, 1601s, 1585w, 1548w, 1523w, 1497w, 1474br, 1440s. δH (d_6-DMSO, 400 MHz) 7.82-7.90 (4H, m), 7.53-7.62 (5H, m), 4.39 (2H, m, 5.42 Hz), 3.75 (2H, s), 3.48 (4H, q, 6.00 Hz), 2.57 (4H, t, 6.32 Hz) ppm. δC (d_6-DMSO, 100 MHz) 152.0 (ArC), 150.9 (ArC), 144.2 (ArC), 131.3 (ArCH), 129.4 (ArCH, 2 overlapped), 122.4 (ArCH), 122.3 (ArCH), 59.2 (CH_2), 58.7 (CH_2), 56.5 (CH_2) ppm. HRMS calculated for $[M + H]^+$: $C_{17}H_{22}N_3O_2{}^+$ 300.1712, found 300.1715.

1.4.3 2-bromo-*N*-(2-bromoethyl)-*N*-(4-(phenyldiazenyl)benzyl)ethan-1-amine

The synthesis was carried out similarly to that in section 1.3.3. 2,2'-((4-(phenyldiazenyl)benzyl)azanediyl)bis(ethan-1-ol) (600 mg, 2.00 mmol) was dissolved in anhydrous DCM (20 mL) and tetrabromomethane (1.46 g, 4.40 mmol) was added, followed by triphenylphosphine (1.16 g, 4.42 mmol). The reaction was stirred at ambient conditions under N_2 for 16 h. The reaction mixture was washed with aqueous *sat.* $NaHCO_3$ x2 and brine x1. The organic phase was dried with anhydrous $MgSO_4$ before removal of solvent by rotary evaporation. Purification was carried out by SiO_2 column chromatography in a mixture of petroleum ether 40-60 and ethyl acetate 6:1 to give the product as an orange oil that crystallised upon standing (715 mg, 84 %). TLC (SiO_2) R_f = 0.62 Pet:EtOAc 6:1. FT-IR ν_{max}/cm^{-1} 3048w, 2969, 2950, 2920, 2871w, 2805br, 2735w, 1602, 1584, 1497w, 1483w, 1461s, 1441s. δH ($CDCl_3$, 400 MHz) 7.87-7.94 (4H, m), 7.45-7.55 (5H, m), 3.82 (2H, s), 3.38 (4H, t, 7.25 Hz), 3.01 (4H, t, 7.25 Hz) ppm. δC ($CDCl_3$, 100 MHz) 152.8 (ArC), 152.3 (ArC), 142.2 (ArC), 131.1 (ArCH), 129.4 (ArCH), 129.2 (ArCH), 123.1 (ArCH), 123.0 (ArCH), 58.8 (CH_2), 56.5 (CH_2), 30.2 (CH_2) ppm. HRMS calculated for $[M + H]^+$: $C_{17}H_{20}N_3Br_2{}^+$ 424.0024, found 424.0007.

1.4.4 1',1"'-(((4-(phenyldiazenyl)benzyl)azanediyl)bis(ethane-2,1-diyl))bis(1-methyl-[4,4'-bipyridine]-1,1'-diium) tetrachloride; AzoV$_2$

The synthesis was carried out similarly to that in section 1.3.4. 1-methyl-[4,4'-bipyridin]-1-ium iodide (856 mg, 2.87 mmol) was dissolved in anhydrous DMF (8 mL) under N_2 at 80 °C. 2-bromo-*N*-(2-bromoethyl)-*N*-(4-(phenyldiazenyl)benzyl)ethan-1-amine (305 mg, 717 μmol) in anhydrous DMF (1 mL) was added in one portion, and the mixture stirred for 3 days at 80 °C. The product precipitant was filtered and washed with further DMF (20 mL) and ACN (40 mL) to give an orange amorphous solid (424 mg). This mixed iodide/bromide salt was dissolved in the minimum volume of water, to which a saturated aqueous solution of ammonium hexafluorophosphate was added until no further precipitation occurred. The product was separated by filtration and dried. The product as a hexafluorophosphate salt was then dissolved in acetone, to which a saturated acetone solution of tetrabutylammonium chloride was added until no further precipitation occurred. The product was filtered off and dried to give an orange amorphous solid (271 mg, 54 %). FT-IR ν_{max}/cm^{-1} 3366br, 3109w, 3000br, 2853w, 1638s, 1560, 1508, 1445. λ_{max}(H_2O)/nm 260 and 332 (ϵ/dm^3 mol^{-1} cm^{-1} 43638 and 19880). δH (D_2O, 500 MHz) 9.04 (4H, d, 6.93 Hz), 8.90 (4H, d, 6.76 Hz), 8.48 (4H, d, 6.88 Hz), 8.44 (4H, d, 6.89 Hz), 7.65-7.73 (5H, m), 7.51 (2H, d, 8.33 Hz), 7.22 (2H, d, 8.33 Hz), 4.92 (4H, t, 6.31 Hz), 4.26 (6H, s), 3.76 (2H, s), 3.56 (4H, t, 6.32 Hz) ppm. δC (D_2O, 125 MHz) 151.3 (ArC), 150.6 (ArC), 149.5 (ArC), 149.0 (ArC), 146.2 (ArCH), 145.6 (ArCH), 142.6 (ArC), 132.4 (ArCH), 130.9 (ArCH), 129.9 (ArCH), 126.5 (ArCH, 2 overlapped), 122.7 (ArCH), 122.2 (ArCH), 59.7 (CH_2), 57.5 (CH_2), 54.3 (CH_2), 48.2 (CH_3) ppm. HRMS calculated for $[M$ - Cl$]^+$: $C_{39}H_{41}N_7{}^{35}Cl_3{}^+$ 712.2484, found 712.2455.

1.5 Np_2V Synthesis

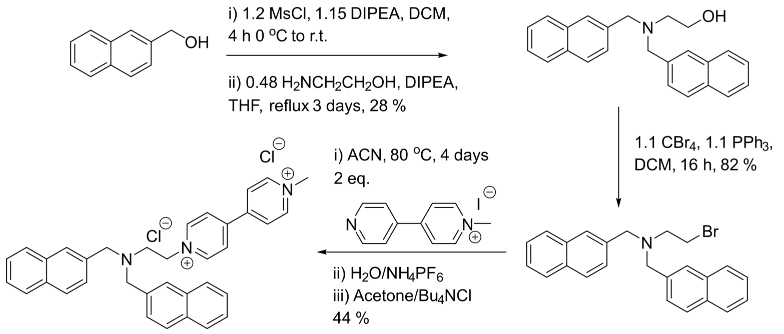

Figure 3: Scheme of Np_2V synthesis.

1.5.1 2-(bis(naphthalen-2-ylmethyl)amino)ethan-1-ol

2-naphthalenemethanol (1.52 g, 9.61 mmol) was dissolved in anhydrous DCM (20 mL) under N_2 and cooled to 0 °C by an ice bath. Methanesulfonyl chloride (0.90 mL, 11.6 mmol) was added, followed by dropwise addition of *N*,*N*-diisopropylethylamine (2.87 mL, 11.1 mmol). The ice bath was removed, and the reaction stirred for 4 h. After dilution with DCM (10 mL), the mixture was washed with saturated NH_4Cl solution twice, dried with anhydrous $MgSO_4$, and the solvent was removed under reduced pressure to give the crude activated alcohol. Ethanolamine (350 mg, 5.73 mmol) was dissolved in anhydrous THF (10 mL) under N_2. The crude activated alcohol was dissolved in anhydrous THF (5 mL), and was added by syringe to the amine followed by dropwise addition of *N*,*N*-diisopropylethylamine (1.29 mL, 4.97 mmol). The mixture was refluxed at 70 °C for 3 days. Solvent was removed under reduced pressure and purification was carried out by SiO_2 column chromatography in a mixture of Pet:EtoAc 2:1 to give the product as a light yellow fluffy solid (543 mg, 33 %). TLC (SiO_2) $R_f = 0.30$ Pet:EtOAc 2:1. FT-IR ν_{max}/cm^{-1} 3446br, 3052, 2973w, 2951s, 2931, 2891, 2872, 2825br, 1634w, 1601s, 1507s, 1480, 1447. δH (d_6-DMSO, 400 MHz) 7.85-7.91 (8H, m), 7.59 (2H, dd, 8.43 and 1.38 Hz), 7.44-7.52 (4H, m), 4.40 (1H, t, 5.38 Hz), 3.80 (4H, s), 3.54 (2H, q, 6.19 Hz), 2.58 (2H, t, 6.61 Hz) ppm. δC (d_6-DMSO, 100 MHz) 137.4 (ArC), 133.0 (ArC), 132.3 (ArC), 127.8 (ArCH), 127.6 (ArCH, 2 overlapped), 127.2 (ArCH), 127.0 (ArCH), 126.1 (ArCH), 125.6 (ArCH), 59.3 (CH_2), 58.5 (CH_2), 55.5 (CH_2) ppm. HRMS calculated for $[M + \text{H}]^+$: $C_{24}H_{24}NO^+$ 342.1852, found 342.1851.

1.5.2 2-bromo-*N*,*N*-bis(naphthalen-2-ylmethyl)ethan-1-amine

2-(bis(naphthalen-2-ylmethyl)amino)ethan-1-ol (249 mg, 729 μmol) was dissolved in anhydrous DCM (10 mL) and tetrabromomethane (267 mg, 805 μmol) was added, followed by triphenylphosphine (211 mg, 804 μmol). The reaction was stirred at ambient conditions under N_2 for 16 h. The reaction mixture was washed with aqueous *sat.* $NaHCO_3$ x2 and brine x1. The organic phase was dried with anhydrous $MgSO_4$ before removal of solvent by rotary evaporation. Purification was carried out by SiO_2 column chromatography in a mixture of petroleum ether 40-60 and ethyl acetate 6:1 to give the product as a light yellow oil that crystallised upon standing (242 mg, 82 %). TLC (SiO_2) $R_f = 0.66$ Pet:EtOAc 6:1. FT-IR ν_{max}/cm^{-1} 3052, 2939, 2800, 2727w, 1632w, 1600s, 1540w, 1506s, 1469w, 1453w, 1439. δH ($CDCl_3$, 500 MHz) 7.83 (6H, d, 8.23 Hz), 7.79 (2H, s), 7.59 (2H, dd, 8.45 and 1.58 Hz), 7.43-7.50 (4H, m), 3.86 (4H, s), 3.40 (2H, t, 7.30 Hz), 2.99 (2H, t, 7.30 Hz) ppm. δC ($CDCl_3$, 125 MHz) 136.8 (ArC), 133.5 (ArC), 133.0 (ArC), 128.2 (ArCH), 127.8 (ArCH, 2 overlapped), 127.5 (ArCH), 127.2 (ArCH), 126.2 (ArCH), 125.8 (ArCH), 59.0 (CH_2), 55.6 (CH_2), 30.4 (CH_2) ppm. HRMS calculated for $[M + H]^+$: $C_{24}H_{23}NBr^+$ 404.1008, found 404.1007.

1.5.3 1-(2-(bis(naphthalen-2-ylmethyl)amino)ethyl)-1'-methyl-[4,4'-bipyridine]-1,1'-diium tetrachloride; Np_2V

1-methyl-[4,4'-bipyridin]-1-ium iodide (375 mg, 1.26 mmol) was dissolved in anhydrous ACN (18 mL) under N_2 at 80 °C. 2-bromo-*N*,*N*-bis(naphthalen-2-ylmethyl)ethan-1-amine (250 mg, 618 μmol) was added in one portion, and the mixture refluxed for 4 days at 80 °C. The product precipitant was filtered and washed with further ACN (40 mL) to give an orange amorphous solid (265 mg). This mixed iodide/bromide salt was dissolved in the minimum volume of 80 °C water, to which a saturated aqueous solution of ammonium hexafluorophosphate was added until no further precipitation occurred. The product was separated by filtration and dried. The product as a hexafluorophosphate salt was then dissolved in acetone, to which a saturated acetone solution of tetrabutylammonium chloride was added until no further precipitation occurred. The product was filtered off and dried to give a light yellow amorphous solid (155 mg, 44 %). FT-IR ν_{max}/cm^{-1} 3351br, 3225w, 3025br, 2832, 2806, 1638s, 1616w, 1598w, 1561, 1508s, 1480w, 1448s. λ_{max}(H_2O)/nm 226 and 265 (ϵ/dm^3 mol^{-1} cm^{-1} 113011 and 24309). δH (D_2O, 500 MHz) 9.00 (2H, d, 6.78 Hz), 8.57 (2H, d, 6.80 Hz), 8.10 (2H, d, 6.78 Hz), 7.80 (2H, d, 6.73 Hz), 7.72 (4H, t, 7.11 Hz), 7.68 (2H, d, 8.40 Hz),

7.52 (2H, s), 7.37-7.44 (4H, m), 7.25 (2H, dd, 8.40 and 1.26 Hz), 4.54 (3H, s), 4.46 (2H, t, 4.72 Hz), 3.70 (4H, s), 3.26 (2H, t, 4.69 Hz) ppm. δC (D_2O, 125 MHz) 147.9 (ArC), 147.8 (ArC), 146.1 (ArCH), 144.9 (ArCH), 136.8 (ArC), 132.8 (ArC), 132.1 (ArC), 128.0 (ArCH), 127.9 (ArCH), 127.8 (ArCH), 127.5 (ArCH, 2 overlapped), 126.5 (ArCH), 126.1 (ArCH), 126.0 (ArCH), 124.9 (ArCH), 60.9 (CH_2), 58.7 (CH_2), 53.9 (CH_2), 48.3 (CH_3) ppm. HRMS calculated for $[M$ - $2Cl]^{2+}$: $C_{35}H_{33}N_3{}^{2+}$ 247.6337, found 247.6335.

1.6 2D NMR

The following section show the 2D NMR spectra for the molecules NpV_2, $AzoV_2$, and Np_2V before and after complexation with CB[7] and/or CB[8]. The assignment of peaks for each complex was carried out as follows. Each peak in the ^{1}H NMR spectrum was correlated to a ^{13}C peak by the HSQC experiment, also noting which HSQC peaks were positive (CH or CH_3) or negative (CH_2). The ^{13}C shifts for each peak were then compared to that of uncomplexed NpV_2 to provide the first evidence for their assignment. The COSY spectrum could then be employed to correlate adjacent protons with each other, allowing further assignment of most of the peaks. The HMBC spectrum was employed last to assign any remaining peaks that had not yet been resolved, and to check over all the previous assignments made by the other techniques. Finally, DOSY NMR confirmed the presence of a single supramolecular species diffusing at a constant value.

1.6.1 $\mathbf{NpV}_2$

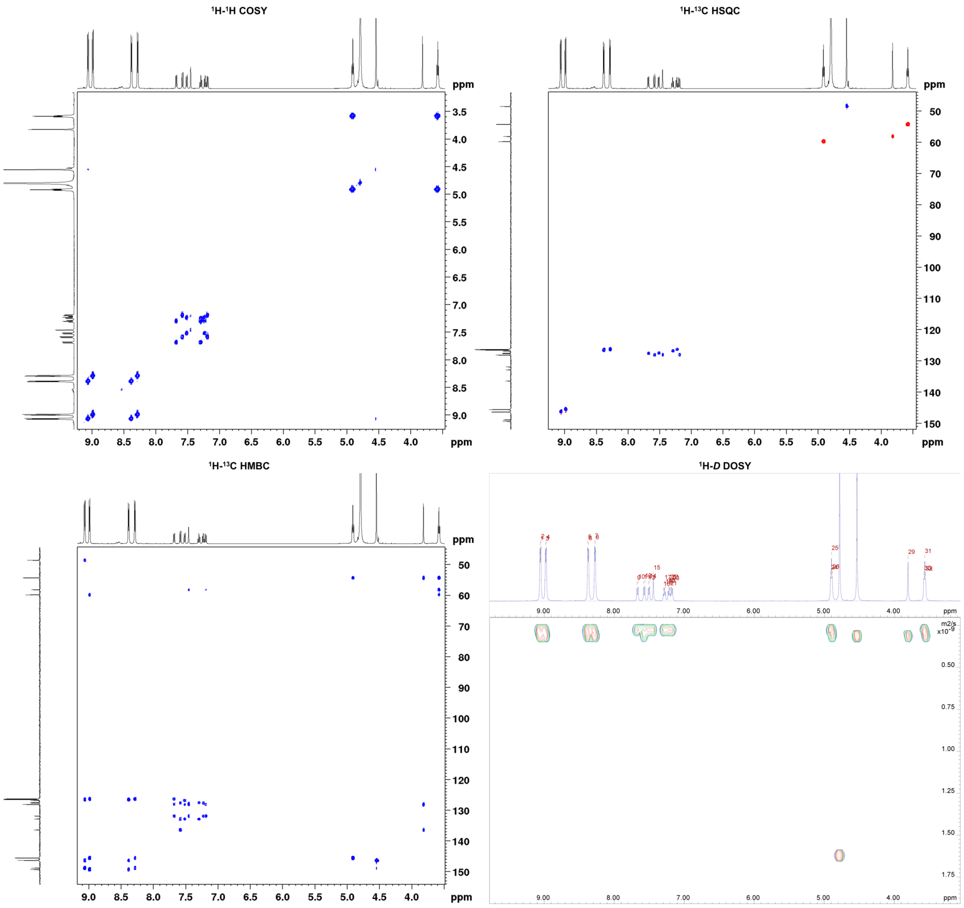

Figure 4: ^{1}H-^{1}H COSY, ^{1}H-^{13}C HSQC, ^{1}H-^{13}C HMBC, and ^{1}H DOSY 2D NMR spectra for $\mathbf{NpV}_2$. For COSY, HSQC and HMBC positive peaks are shown in blue and negative peaks are shown in red.

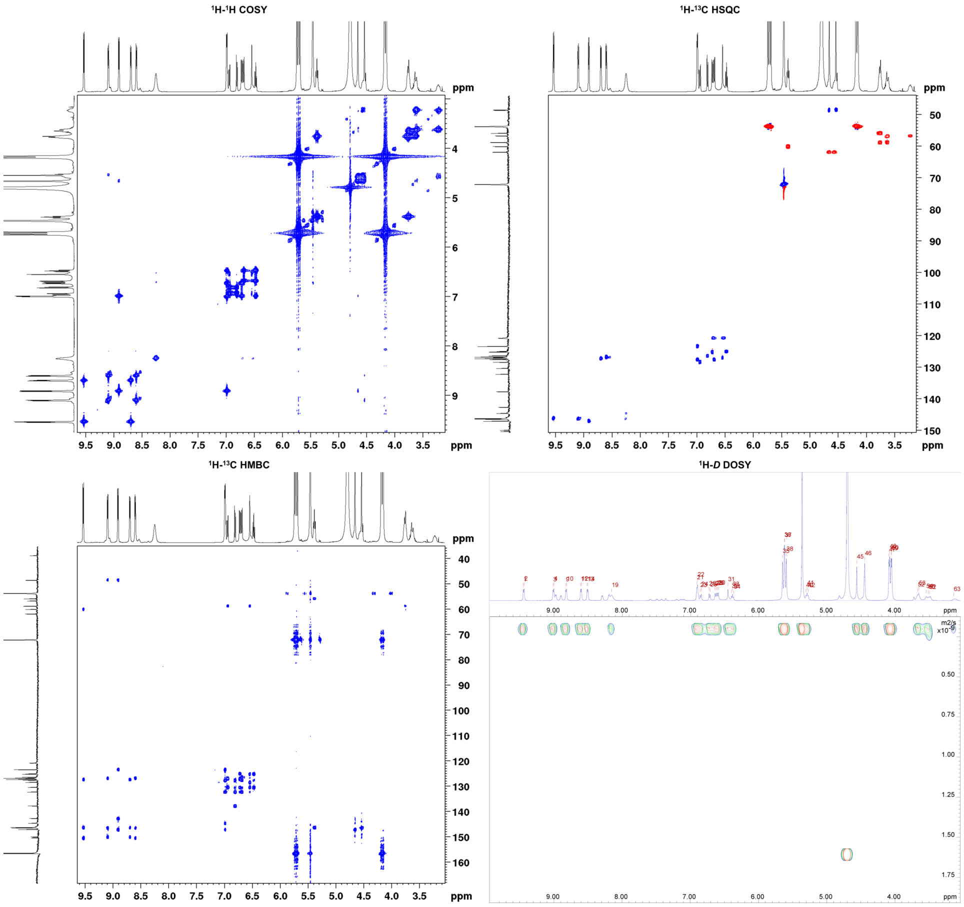

Figure 5: ^{1}H-^{1}H COSY, ^{1}H-^{13}C HSQC, ^{1}H-^{13}C HMBC, and ^{1}H DOSY 2D NMR spectra for the **NpV$_2$·CB[8]** complex. For COSY, HSQC and HMBC positive peaks are shown in blue and negative peaks are shown in red.

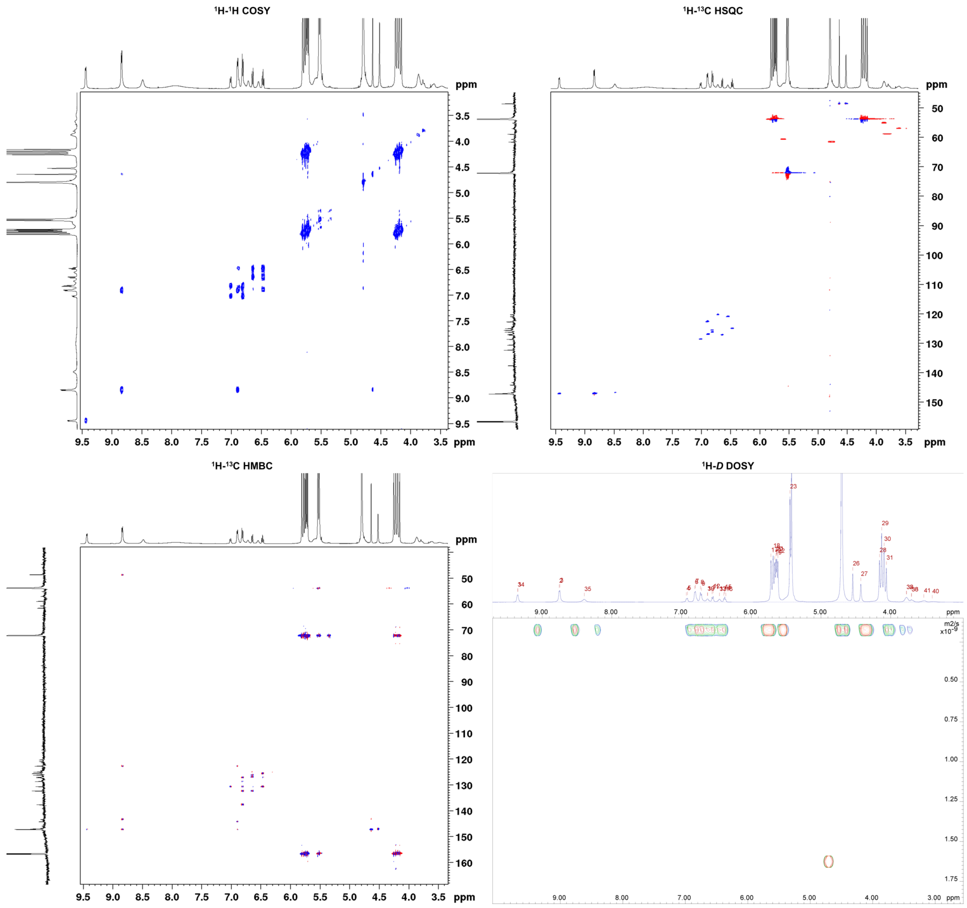

Figure 6: ^{1}H-^{1}H COSY, ^{1}H-^{13}C HSQC, ^{1}H-^{13}C HMBC, and ^{1}H DOSY 2D NMR spectra for the $\mathbf{NpV}_2\cdot(\mathbf{CB[8]})_2$ complex. For COSY, HSQC and HMBC positive peaks are shown in blue and negative peaks are shown in red.

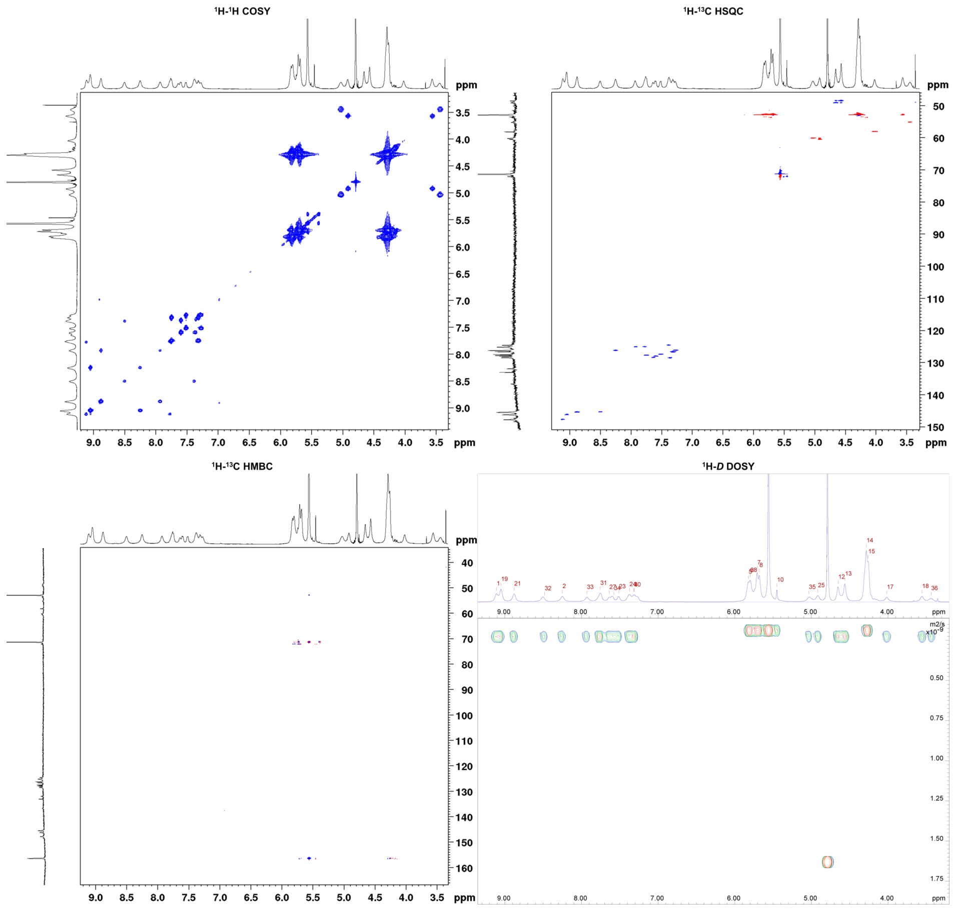

Figure 7: ^{1}H-^{1}H COSY, ^{1}H-^{13}C HSQC, ^{1}H-^{13}C HMBC, and ^{1}H DOSY 2D NMR spectra for the **NpV$_2$·CB[7]** complex. For COSY, HSQC and HMBC positive peaks are shown in blue and negative peaks are shown in red.

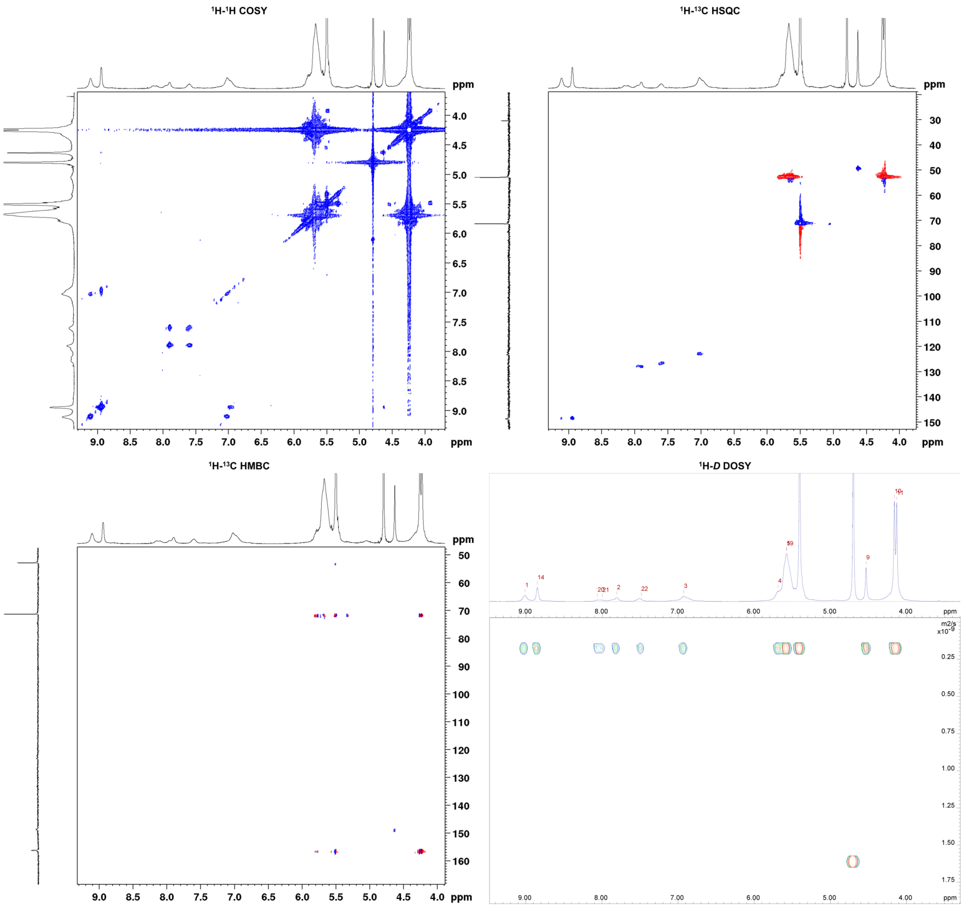

Figure 8: ^{1}H-^{1}H COSY, ^{1}H-^{13}C HSQC, ^{1}H-^{13}C HMBC, and ^{1}H DOSY 2D NMR spectra for the $\mathbf{NpV_2{\cdot}(CB[7])_2}$ complex. For COSY, HSQC and HMBC positive peaks are shown in blue and negative peaks are shown in red.

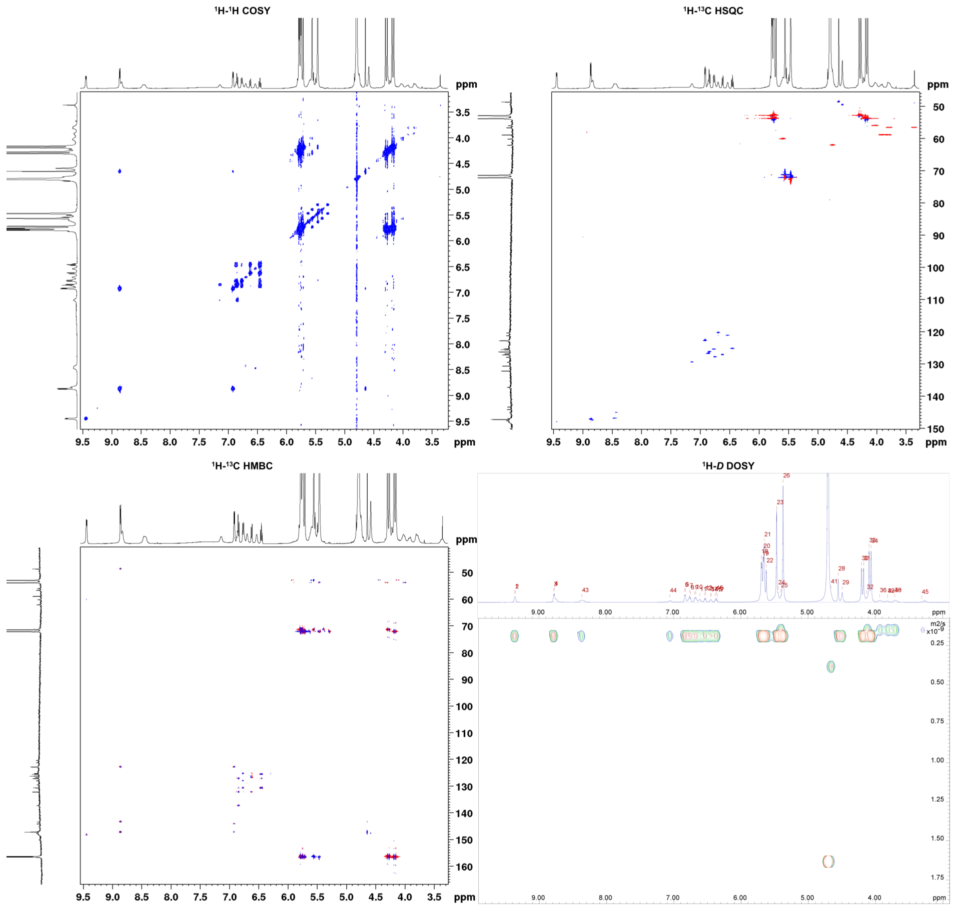

Figure 9: ^{1}H-^{1}H COSY, ^{1}H-^{13}C HSQC, ^{1}H-^{13}C HMBC, and ^{1}H DOSY 2D NMR spectra for the **NpV**$_2$·**(CB[8])(CB[7])** complex. For COSY, HSQC and HMBC positive peaks are shown in blue and negative peaks are shown in red.

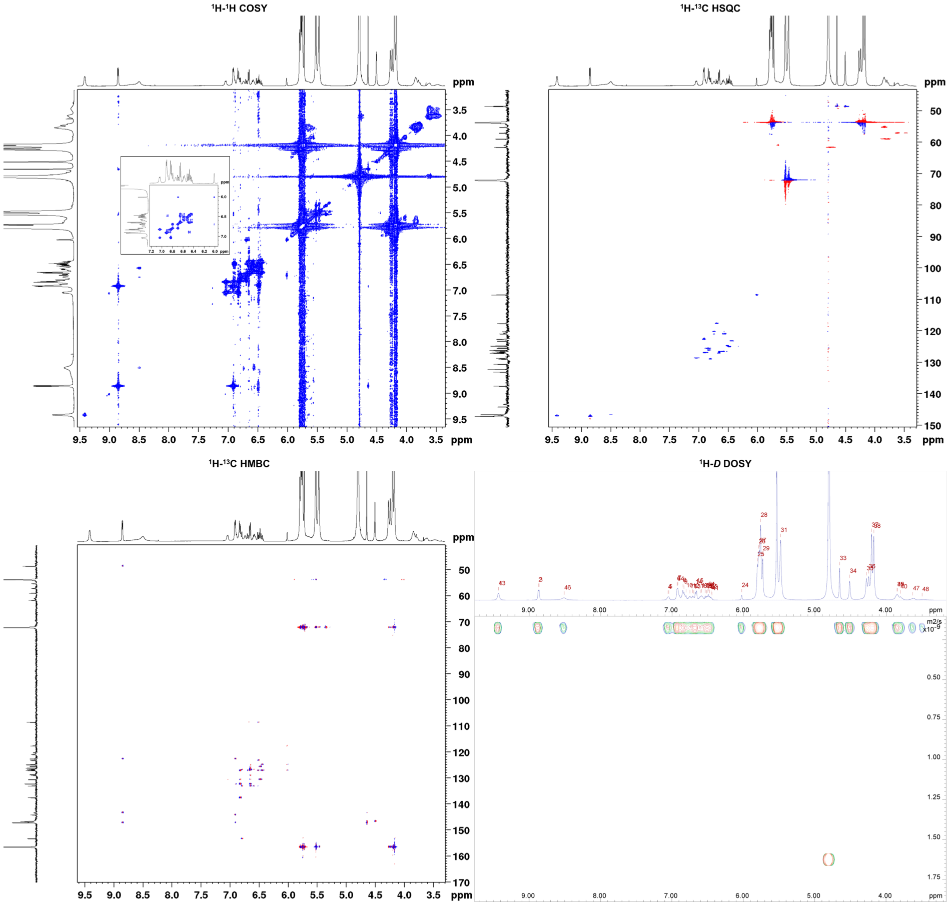

Figure 10: ^{1}H-^{1}H COSY, ^{1}H-^{13}C HSQC, ^{1}H-^{13}C HMBC, and ^{1}H DOSY 2D NMR spectra for the **NpV$_2$·(CB[8])$_2$ and 2-naphthol** complex. For COSY, HSQC and HMBC positive peaks are shown in blue and negative peaks are shown in red.

1.6.2 AzoV$_2$

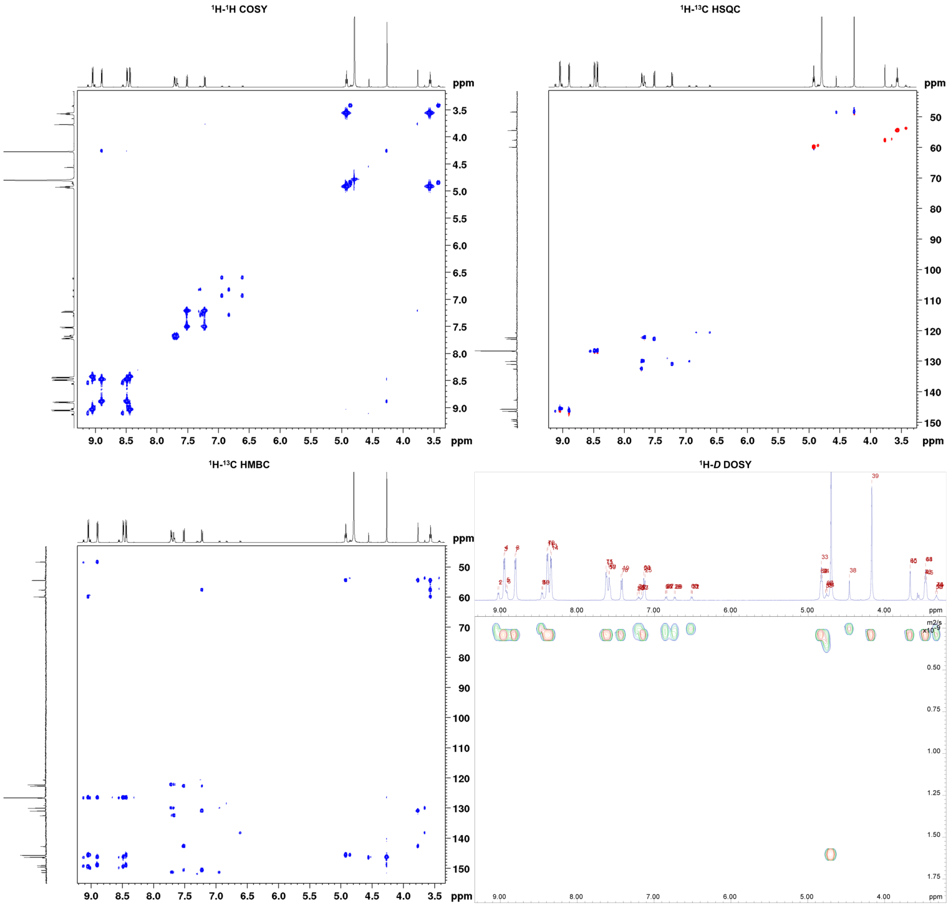

Figure 11: ^{1}H-^{1}H COSY, ^{1}H-^{13}C HSQC, ^{1}H-^{13}C HMBC, and ^{1}H DOSY 2D NMR spectra for **AzoV**$_2$. For COSY, HSQC and HMBC positive peaks are shown in blue and negative peaks are shown in red.

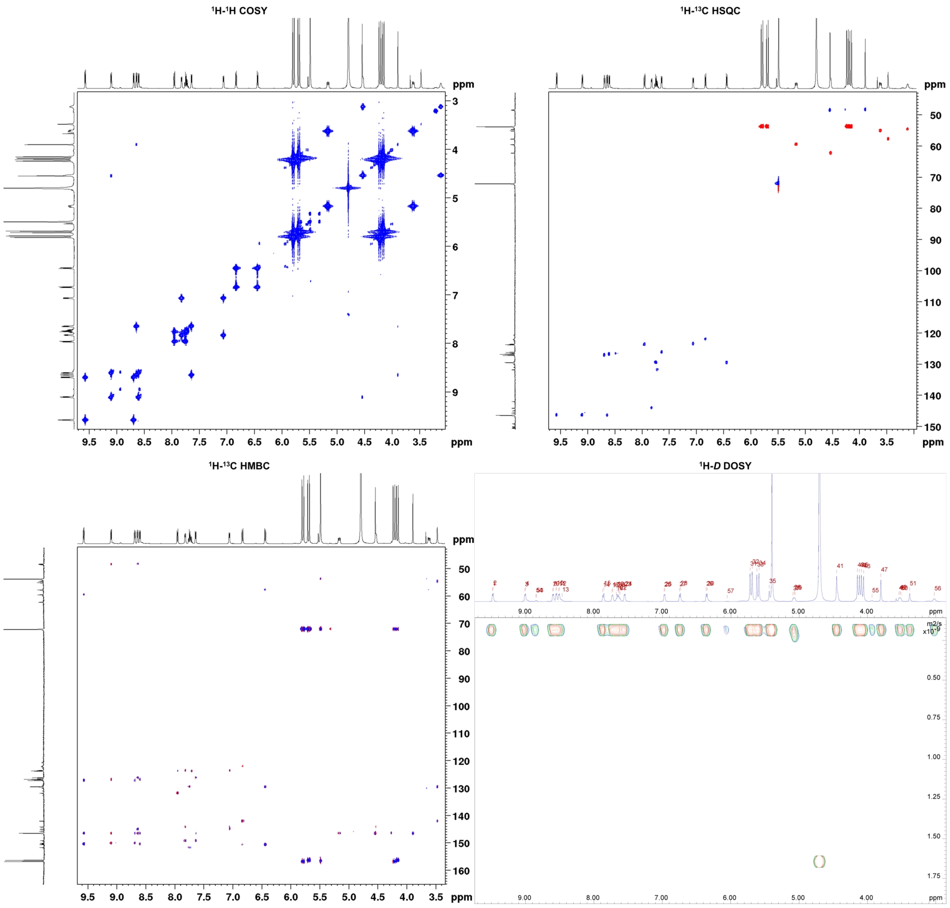

Figure 12: ^{1}H-^{1}H COSY, ^{1}H-^{13}C HSQC, ^{1}H-^{13}C HMBC, and ^{1}H DOSY 2D NMR spectra for the **AzoV**$_2$·**CB[8]** complex. For COSY, HSQC and HMBC positive peaks are shown in blue and negative peaks are shown in red.

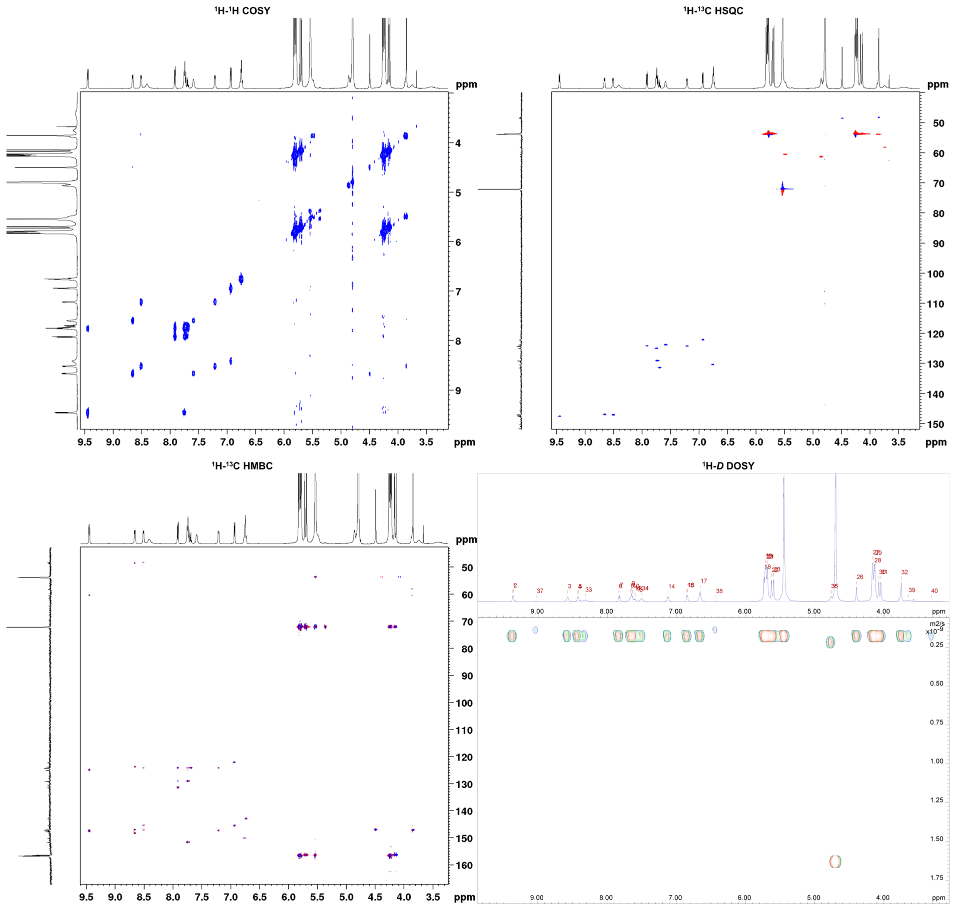

Figure 13: ^{1}H-^{1}H COSY, ^{1}H-^{13}C HSQC, ^{1}H-^{13}C HMBC, and ^{1}H DOSY 2D NMR spectra for the **AzoV**$_2$·(**CB[8]**)$_2$ complex. For COSY, HSQC and HMBC positive peaks are shown in blue and negative peaks are shown in red.

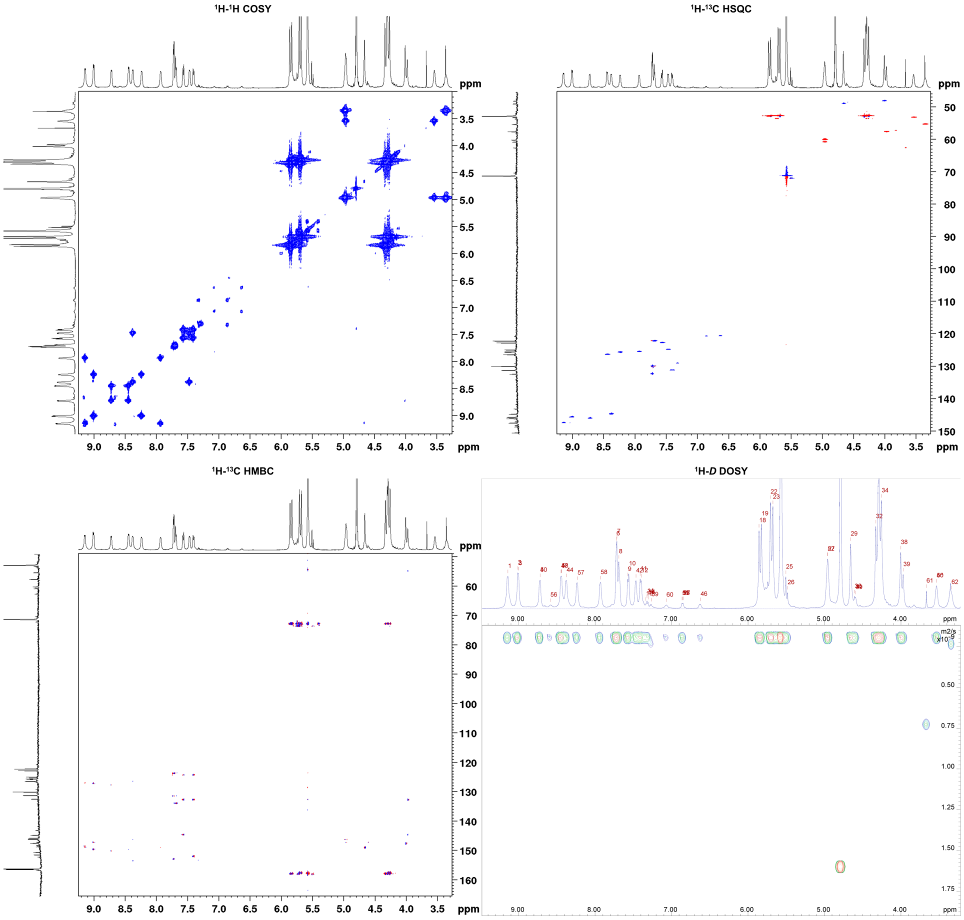

Figure 14: ^{1}H-^{1}H COSY, ^{1}H-^{13}C HSQC, ^{1}H-^{13}C HMBC, and ^{1}H DOSY 2D NMR spectra for the **AzoV$_2$·CB[7]** complex. For COSY, HSQC and HMBC positive peaks are shown in blue and negative peaks are shown in red.

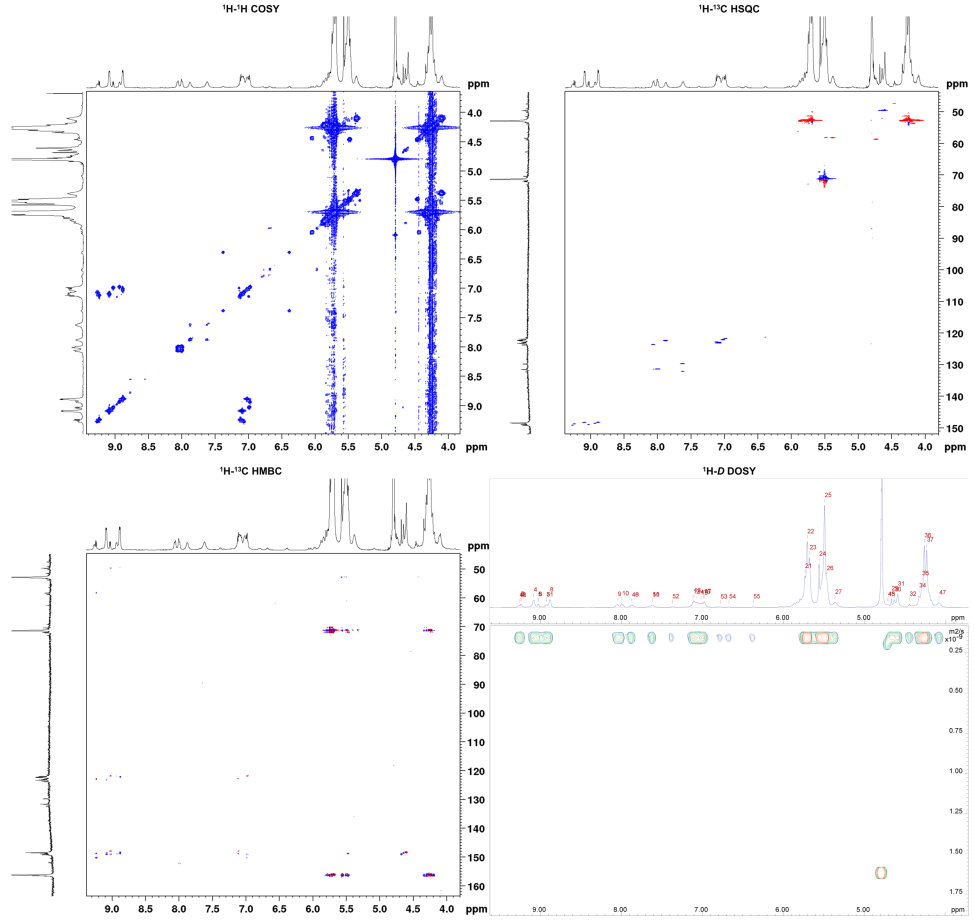

Figure 15: ^{1}H-^{1}H COSY, ^{1}H-^{13}C HSQC, ^{1}H-^{13}C HMBC, and ^{1}H DOSY 2D NMR spectra for the **AzoV**$_2$·**(CB[7])**$_2$ complex. For COSY, HSQC and HMBC positive peaks are shown in blue and negative peaks are shown in red.

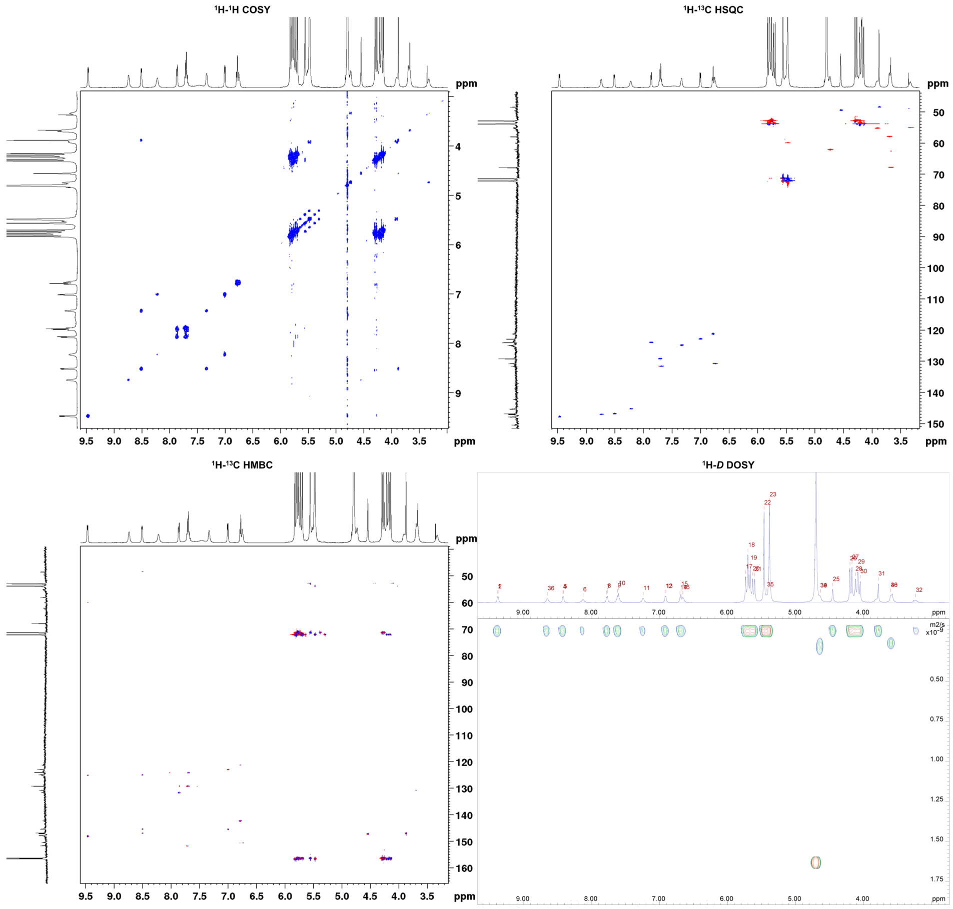

Figure 16: ^{1}H-^{1}H COSY, ^{1}H-^{13}C HSQC, ^{1}H-^{13}C HMBC, and ^{1}H DOSY 2D NMR spectra for the **AzoV**$_2$·**(CB[8])(CB[7])** complex. For COSY, HSQC and HMBC positive peaks are shown in blue and negative peaks are shown in red.

1.6.3 $\mathbf{Np_2V}$

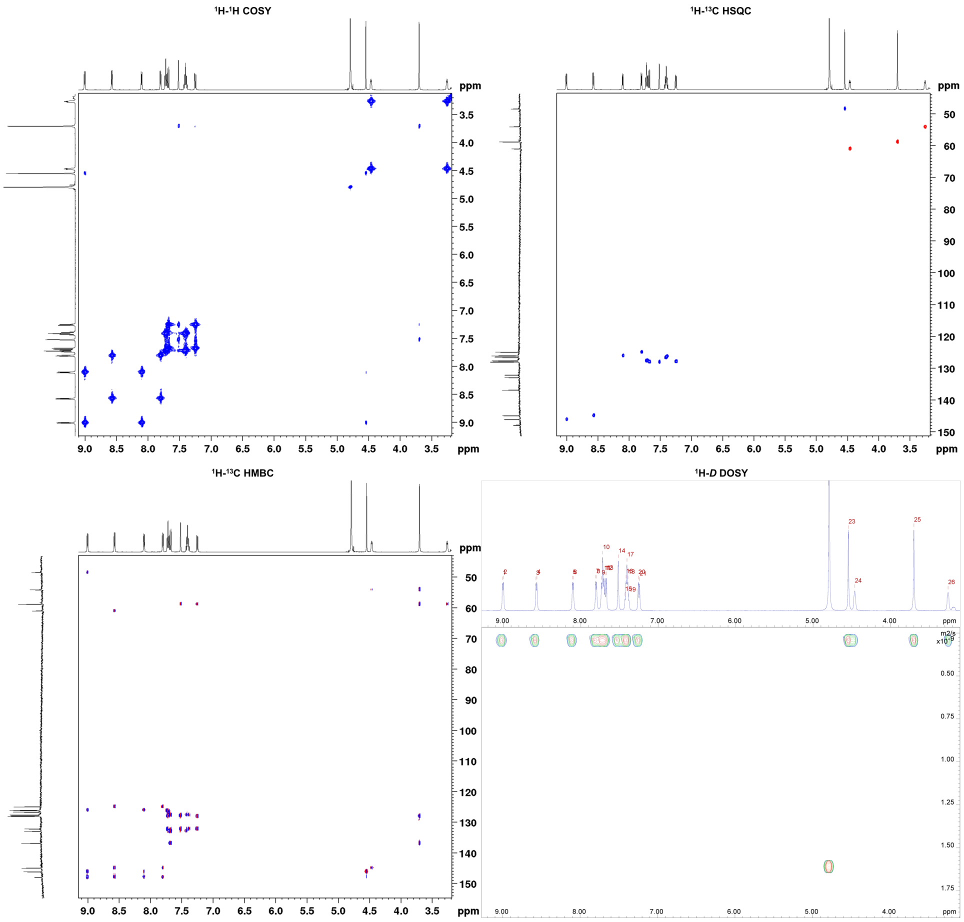

Figure 17: ^{1}H-^{1}H COSY, ^{1}H-^{13}C HSQC, ^{1}H-^{13}C HMBC, and ^{1}H DOSY 2D NMR spectra for $\mathbf{Np_2V}$. For COSY, HSQC and HMBC positive peaks are shown in blue and negative peaks are shown in red.

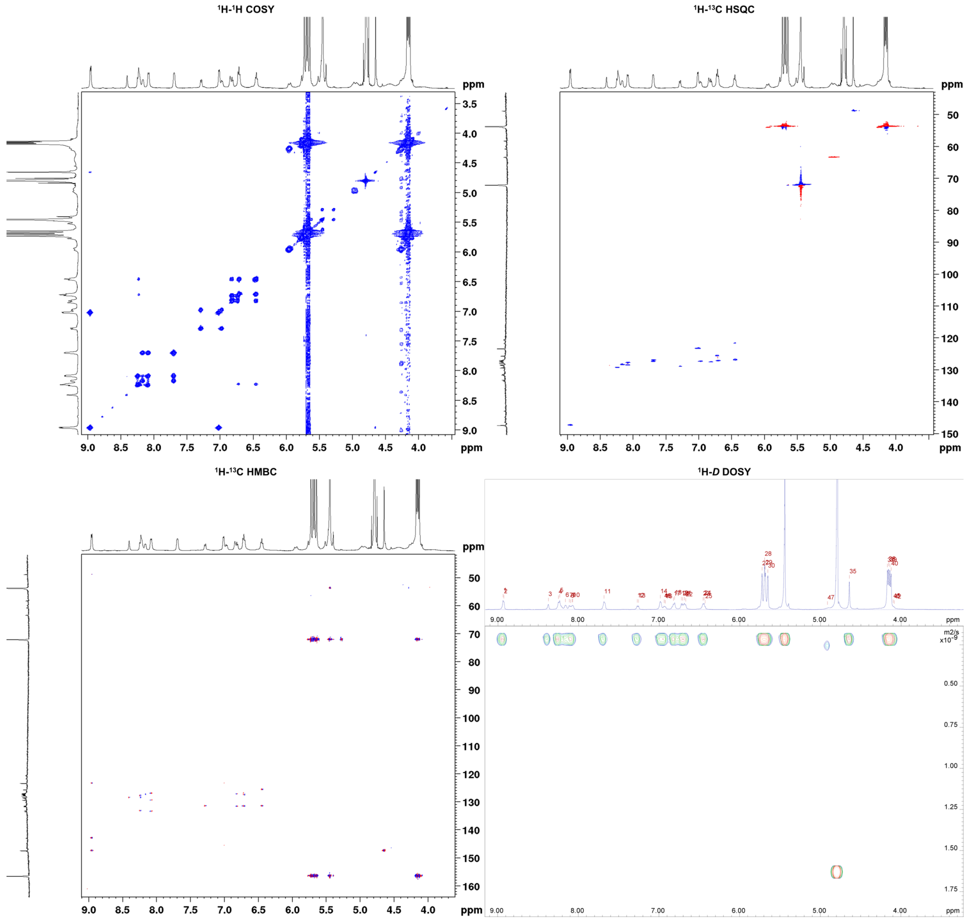

Figure 18: ^{1}H-^{1}H COSY, ^{1}H-^{13}C HSQC, ^{1}H-^{13}C HMBC, and ^{1}H DOSY 2D NMR spectra for the $\mathbf{Np_2V{\cdot}CB[8]}$ complex. For COSY, HSQC and HMBC positive peaks are shown in blue and negative peaks are shown in red.

1.7 ^1H NMR Titrations

1.7.1 NpV_2-CB[8]

In Fig. 2 in the main text of the manuscript it was observed that all of the protons corresponding to the Np group have shifted significantly upfield meaning they are complexed within the CB[8] cavity. As there are two equivalent V environments present in NpV_2, when one CB[8] is complexed asymmetry is introduced to the molecule. The peaks corresponding to the V complexed with Np show shifts upfield, and those uncomplexed show shifts downfield. It is possible to derive the location of the CB[8] on the molecule by the degree of shifting observed for different environments. For example, peak *a* showed a downfield shift upon complexation, whereas peak *b* showed a slight upfield shift. We can determine that the CB[8] must therefore encapsulate protons in environment *b*, but *a* is just outside the cavity. Peaks *d* and *c* show very large upfield shifts of 1.8 and 1.4 ppm, showing they are far inside the cavity. Peak *e* then has a smaller upfield shift, and also displays some broadening; evidencing dynamic binding in this region. Peaks *f*, *g*, and *h* reflect this hypothesis, by showing they are also encapsulated in the cavity are more dynamic, due to their higher degrees of conformational freedom. The effect of downfield shifting on the unbound V environments decayed with distance from the CB[8] carbonyl portals, with the effect greatest for *e* and smallest for *b*.

After the addition of a further CB[8], the second V present in the molecule will form a 1:1 binary complex. This was more dynamic, due to the weaker binding strength. The Np protons remain upfield shifted as they are bound to CB[8] in the intramolecular complex. The remaining V now binds dynamically to CB[8], but only from environments *a* - *d*, with *e*, *f* and *g* showing downfield shifts. An additional CB[8] environment was also observed, but this extra peak did not display any splitting. This means the second CB[8] had fast dissociation kinetics. Overall this shows that intramolecular host-guest binding takes precedent. Molecular mechanics optimisation also provide evidence for the proposed binding, shown in Fig. 3 in the main text.

1.7.2 NpV_2-CB[7]

Titration of NpV_2 with CB[7], which can only encapsulate one guest molecule as opposed to two, was carried out. Upon complexation with 1 CB[7], similar changes in chemical shift are observed as for with CB[8] but with much broader peaks. This broadness was attributed to a smaller overall bind-

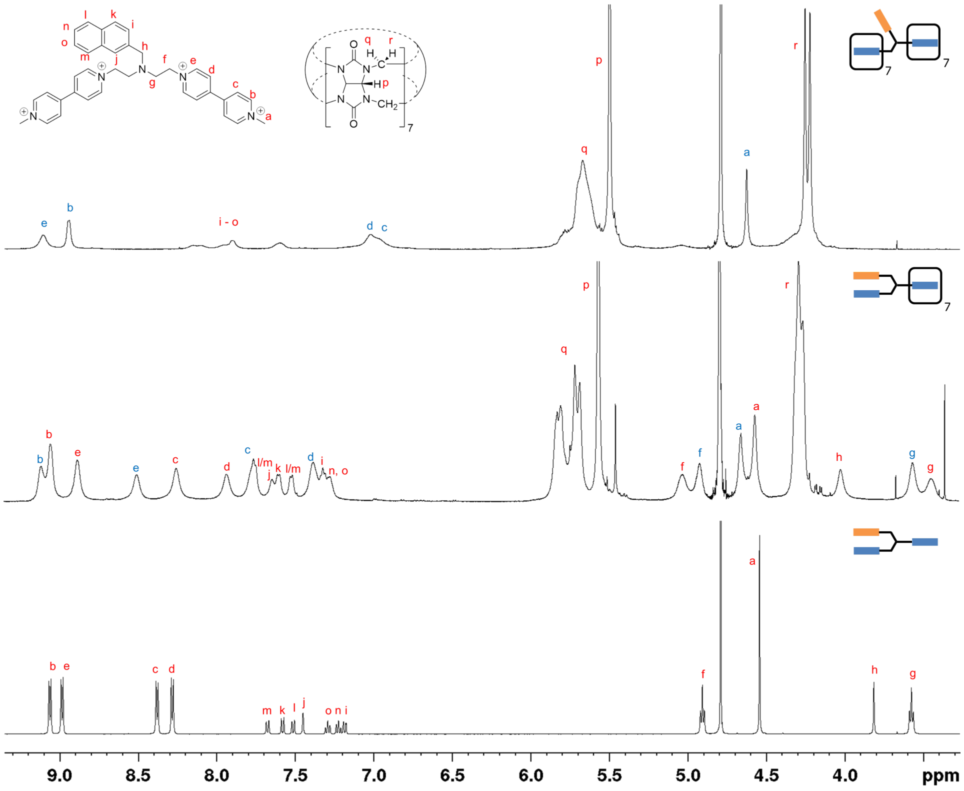

Figure 19: Stacked ^{1}H NMR spectra of NpV_2 in D_2O (bottom spectrum), followed by addition of 1.0 (middle spectrum) and then 2.0 *equiv.* of CB[7] (top spectrum). The peak assignments in red show proton environments outside of the CB[7] cavity, and those in blue are inside the cavity.

ing affinity (typically $K_a = 10^{4-6}$ M^{-1}),[5] and some motion of the CB[7] occurring along the molecule. This results in averaging the chemical environment as it is in partial flux, but predominately in a single bound state. The complexed V peaks c, d, e exhibit significant upfield shifts, more so for c and d, allowing the derivation of the position of CB[7]. Peaks a and b show slight downfield shifts, meaning these environments are just outside the cavity. In contrast to complexation with CB[8], peaks f and g remain unchanged in their chemical shifts, likely a result of the smaller CB[7] cavity size not allowing their partial complexation. A further result of complexation is the remaining uncomplexed V became conformationally confined adjacent to the free Np group. As a result of the Np group's aromatic ring

current, the nearby protons *e*, *f*, *c*, *d*, *h* are relatively more shielded and show slight upfield shifts. Equally a downfield shift can be observed for protons *g* just outside the effect of the ring current. Splitting of the broad CB[7] peak *q* was observed, but was not obvious for peak *r*. This is a result of the protons facing towards the CB[7] experiencing more of a local difference in chemical environment than those facing away between the top and bottom of the CB[7]. The Np peaks also demonstrate a partial downfield shift as a result of proximity to the carbonyl portals of the complexed CB[7], more obvious for peaks *i*, *j*, and *k*.

Upon complexation of a further CB[7] molecule, the spectrum became more dynamic in its binding, with very broad peaks. Key information could still be elucidated, however, such as the loss of asymmetry in the binding resulting in a single peak for the V protons *a* - *e* that could easily be assigned. Peaks *a* and *e* were partially downfield shifted, whereas *b*, *c*, and *d* were upfield shifted to different extents. This allowed the CB[7] position to be derived as fully encapsulating protons *c* and *d* with partial encapsulation of *b*. The Np peaks *l* - *o* showed a further downfield shift as compared to when complexing one CB[7], further supporting the proposed structure as this group would be adjacent to the carbonyl portals of two CB[7] molecules. The sp^3 protons *f*, *g* and *h* were in highly dynamic states and could not be observed clearly. Overall this allows the model in Fig. 20 to be proposed for the binding architecture.

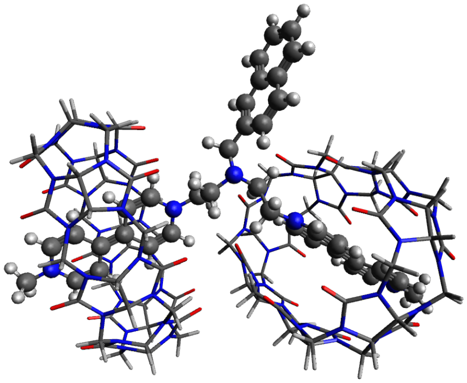

Figure 20: The proposed complex of NpV_2 with 2 CB[7] molecules forming a symmetric complex. The positions of CB[7] are derived from the ^1H NMR experiments and optimisation was carried out with MMFF94 molecular mechanics in Avogadro v1.2.

1.7.3 $AzoV_2$-CB[8]

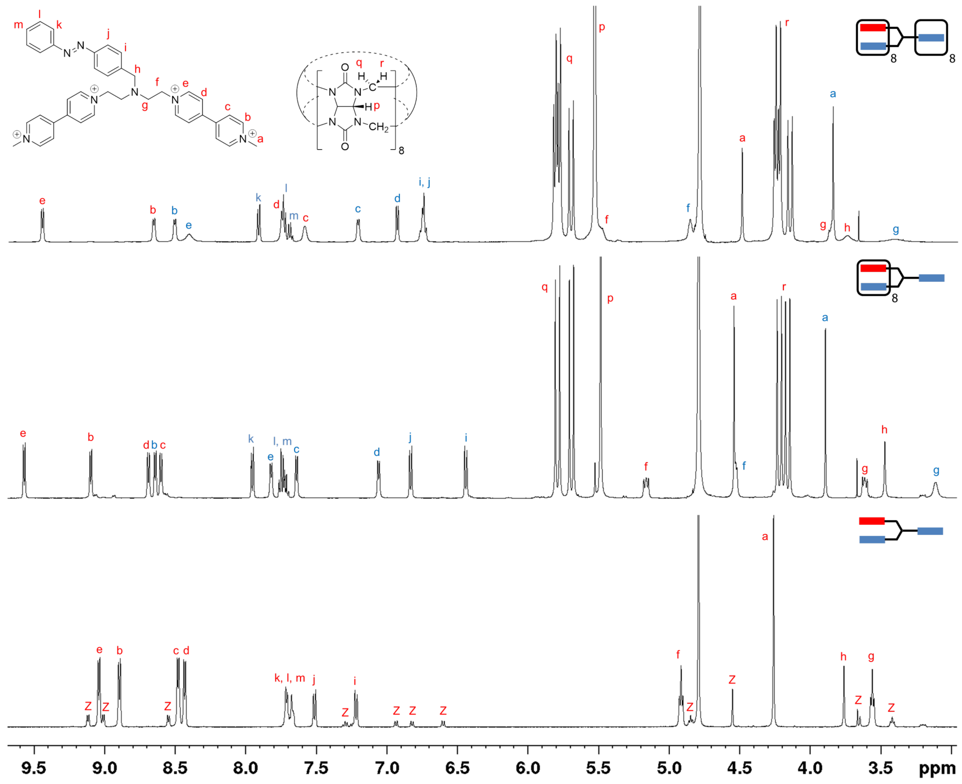

Figure 21: Stacked ^1H NMR spectra of $AzoV_2$ in D_2O (bottom spectrum), followed by addition of 1.0 (middle spectrum) and then 2.0 *equiv.* of CB[8] (top spectrum). The peak assignments in red show proton environments outside of the CB[8] cavity or forming a 1:1 binary complex with viologen, and those in blue are inside the cavity as an intramolecular complex.

Upon introduction of 1 CB[8], complexation of one V moiety and partial encapsulation of the Azo was observed. Asymmetry in the molecule has again been introduced, with two sets of peaks corresponding to each V moiety present. The V aromatic peaks *c*, *d*, and *e* show a significant upfield shift of more than 1 ppm, and *b* shows a smaller shift of 0.3 ppm. This shows the CB[8] cavity fully encapsulates *c*, *d*, and *e* with partial encapsulation of *b*. In comparison to the binary complex between NpV_2 and 1 CB[8], peak *a* also shows an upfield shift of almost 0.4 ppm. This was thought to not be due to encapsulation in CB[8], instead to be due to the extended length

of the azobenzene, allowing an overlap of the aromatic ring current with protons a (ring k - m), causing a shielding effect. Peaks f and g also show encapsulation within the cavity and some broadening similar to NpV_2. The first aromatic ring of the azobenzene (i and j) shows large upfield shifts, with k showing downfield shifts and l and m remaining unchanged. This implies the CB[8] can only encapsulate one ring of the Azo. The CB[8] peaks q and r show splitting as for NpV_2, implying the complex formed is strong with slow dissociation kinetics.

With a further CB[8] molecule, a similar asymmetric intramolecular complex can be observed as for NpV_2, with both a heteroternary complex and a binary complex present. This is evidenced from the changes in peaks of the previously uncomplexed V, where b, c, d (in red) show upfield shifts, and e, f, a have shifted downfield. Changes to the peaks for the heteroternary complex are also observed, with most significantly the peak e (in blue) is shifted much less upfield compared to before, likely a result of the adjacent second CB[8] making complexation close to the core of the molecule less favourable. The second CB[8] peaks q and r do not show splitting again showing how this second interaction is less strong. This allowed the model in Fig. 22 to be proposed.

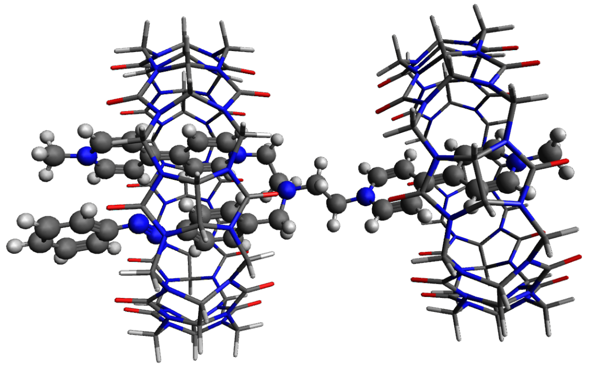

Figure 22: The proposed complex of $AzoV_2$ with 2 CB[8] molecules forming an asymmetric complex. The positions of CB[8] are derived from the ^{1}H NMR experiments and optimisation was carried out with MMFF94 molecular mechanics in Avogadro v1.2.

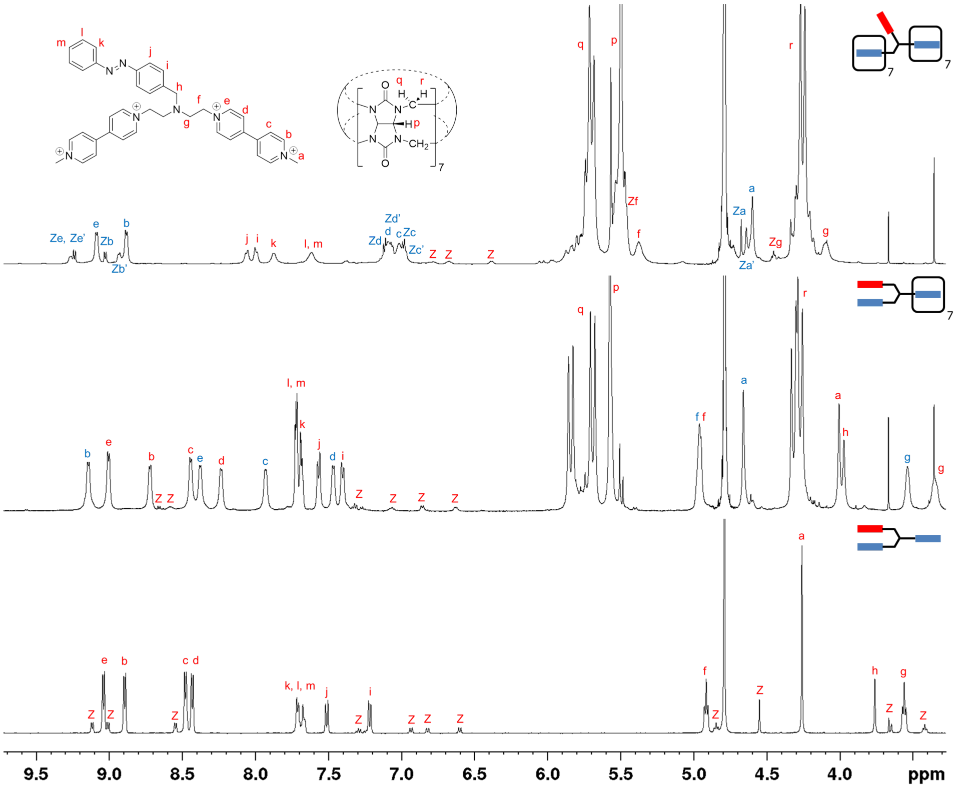

Figure 23: Stacked ^{1}H NMR spectra of AzoV$_2$ in D$_2$O (bottom spectrum), followed by addition of 1.0 (middle spectrum) and then 2.0 *equiv.* of CB[7] (top spectrum). The peak assignments in red show proton environments outside of the CB[7] cavity, and those in blue are inside the cavity. Two different complexes with the Z isomer could be observed, denoted Zx and Zx' where applicable.

1.7.4 AzoV$_2$-CB[7]

Upon introduction of a CB[7] host, one V was encapsulated in its cavity as for NpV$_2$ however in this case the peaks were sharper. This implied there was a more stable equilibrium state. The complexed V peaks c, d, e exhibit significant upfield shifts being inside the cavity, with b and a showing downfield shifts. The peaks f and g adjacent to CB[7] were unchanged in their chemical shift as for NpV$_2$. As a result of complexation, the Azo is now conformationally confined adjacent to the remaining V, allowing interactions of their ring currents to affect relative shielding from the magnetic field. This

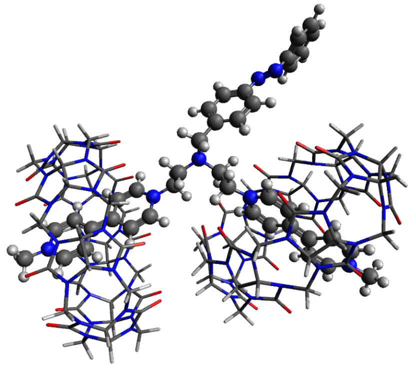

Figure 24: The proposed complex of $AzoV_2$ with 2 CB[7] molecules forming a symmetric complex. The positions of CB[7] are derived from the ^1H NMR experiments and optimisation was carried out with MMFF94 molecular mechanics in Avogadro v1.2. Azobenzene being twisted or planar in polar solvents is still under debate.[6]

was mostly observed for peaks *a*, *b*, *d* and *g*, which correlates with the azo bond separation between the two aromatic rings of the Azo. Splitting of both CB[7] peaks *q* and *r* was observed, evidencing this was also a 'locked' complex. The Azo protons remained almost unchanged after complexation, with only a slight downfield shift observed for protons *i*, which are the closest to the bound CB[7] carbonyl portals.

Upon introduction of a further CB[7], a complex spectrum was obtained. The spectrum was less dynamic and more resolved than for the case with NpV_2 likely due to the more extended Azo less affected by confinement between the two CB[7] molecules. The dominant *E* isomer complex could be readily observed, with symmetry being recovered and so a single set of peaks shown for the V protons *a* - *g*. Peaks *a* and *e* were downfield shifted, whereas *b*, *c*, and *d* were upfield shifted to different extents. This allowed the CB[7] position to be derived as fully encapsulating protons *c* and *d* with partial encapsulation of *b*. The Azo protons *i*, *j*, and *k* showed significant downfield shifting being in close proximity to two sets of CB[7] cabronyl portals, with *l* and *m* remaining relatively unchanged. Peaks *f* and *g* showed significant downfield shifts also expected from proximity to the portals, with *h* becoming too broad to observe.

A subset of peaks was also observed relating to the *Z* isomer in two different configurations. As the Azo moiety is not encapsulated within the

cavity, evidenced by its protons experiencing significant downfield shifting, it will be present in equilibrium between the *E* and *Z* isomer. Two clear sets of peaks for the viologen protons *a* - *e* was observed in similar regions as for the *E* isomer. This could be a result of the introduction of asymmetry in the complex due to the presence of the *Z* isomer, causing steric crowding on perhaps one side of the molecule. However, this would require quite a kinetically trapped complex to prevent averaging of the peaks in the ^{1}H NMR. A more likely reason would be the presence of two equilibrium states with similar energy minima in competition. This is supported by further experiments into the photo-isomerisation of these complexes as discussed in the main text, where after UVA exposure to drive the formation of the *Z* isomer, a single *Z* isomeric state dominates as opposed to an asymmetric complex. This allowed the model in Fig. 24 to be proposed for the *E* isomer.

1.7.5 AzoV$_2$-CB[8]-CB[7]

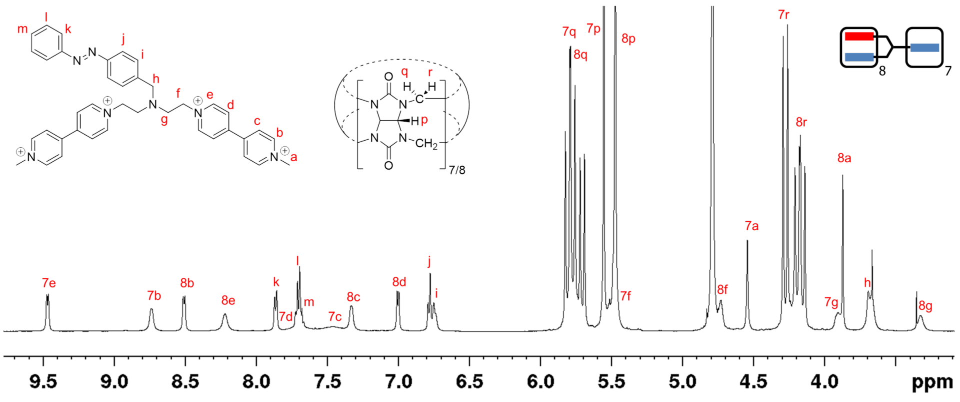

Figure 25: ^{1}H NMR spectrum of AzoV$_2$ in D_2O with 1 *equiv.* of CB[8] and of CB[7]. The initially symmetric molecule is now asymmetric, so peaks corresponding to complexation with CB[7] or CB[8] can be assigned with the prefix 7 or 8 respectively.

A mixed CB[7] and CB[8] complex was also prepared as for NpV$_2$, with spectrum shown in Fig. 25. Here we can see similarities to binding 1 CB[8] or 1 CB[7] to AzoV$_2$. For example, the largest upfield shifts for the Azo moiety were observed for protons *i* and *j*, with *k* - *m* extended beyond the CB[8] cavity. Equally, the largest shifts for the V moieties upon complexation of either CB[7] or CB[8] were observed for protons *c* and *d*, evidencing this is the predominant location of the CB cavities. The alkyl units *f* and *g* were

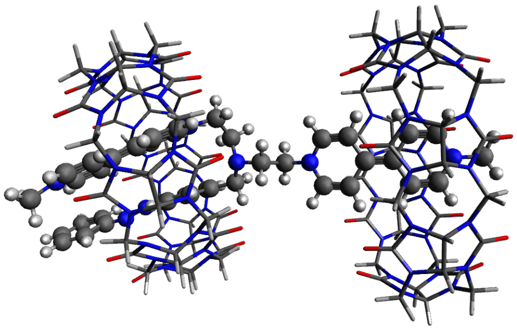

Figure 26: The proposed complex of $AzoV_2$ with CB[8] and CB[7] molecules forming a self-sorting complex. The positions of CB[7] and CB[8] are derived from the ^{1}H NMR experiments and optimisation was carried out with MMFF94 molecular mechanics in Avogadro v1.2.

only within the cavity for CB[8], showing upfield shifts, whereas downfield shifts are observed with CB[7]. Similar to the case with NpV_2, splitting of the peaks *q* and *r* was only observed for the CB[8] complex. A calculated model is shown in Fig. 26.

1.7.6 Np_2V-CB[8]

Upon introduction of CB[8] to Np_2V a similar type of intramolecular complexation can be observed (Fig. 27). The NMR spectrum is more complex however, due to the presence of two Np groups which have very similar proton and carbon chemical shift, making spectrum interpretation more difficult. All of the aromatic peaks for V show a marked upfield shift, with the largest shifts present for *c* and *d*, and smaller for *b* and *e*. Peaks *a* and *f* show downfield shifts, so the CB[8] cavity must be located in the centre of the V mainly over protons *c* and *d*. Peaks *h* and *g* have become too broad to enable assignment through ^{1}H-^{1}H or ^{1}H-^{13}C correlation spectra, showing they have become highly dynamic in their environment. One of the Np groups has also been encapsulated in CB[8], introducing asymmetry in the complex. Peaks (in blue) *i*, *j*, and *k* show smaller upfield shifts than *l* - *o*, implying the centre of the cavity sits more over the end of the Np group. This is more obvious from the molecular model proposed in Fig. 28. The unbound Np group now shows significant downfield shifting for peaks *i*, *j*,

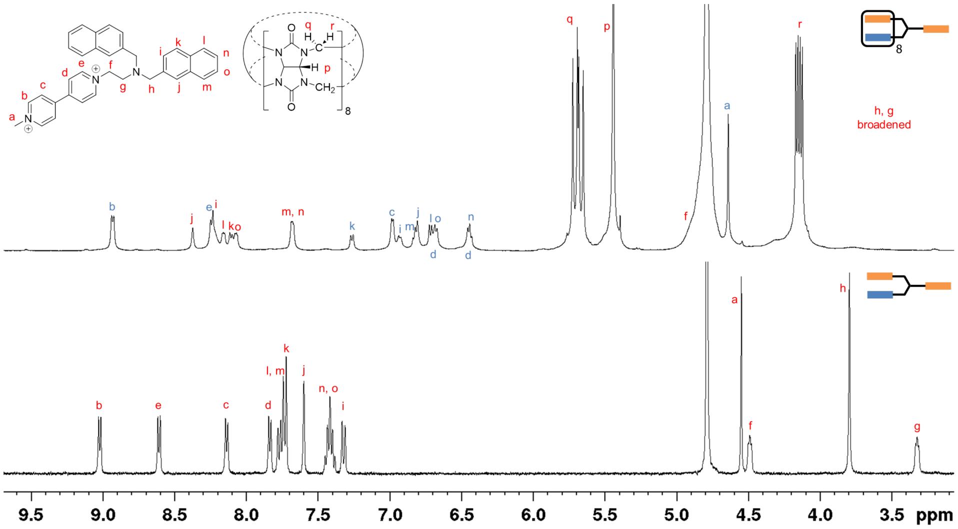

Figure 27: ^{1}H NMR spectrum of Np_2V in D_2O with 1 *equiv.* of CB[8]. The initially symmetric molecule is now asymmetric.

k, *l*, and *o*, more so than would be expected for simply being in proximity to the carbonyl portals of CB[8]. Peak *n* shows a smaller downfield shift, and peak *m* shows a very slight upfield shift of 0.05 ppm. This was thought to be due to the unbound hydrophobic Np having very unfavourable interactions with the solvent, and so will be conformationally confined as close to the external surface of the CB[8] as possible. This results in more of the protons being in quite close proximity to the carbonyl portals, resulting in downfield shifts. Peak *m* could be displaying a slight upfield shift due to being just outside the effect of the carbonyl, similar to the aromatic ring currents discussed previously, resulting in being more shielded. The CB[8] protons *q* and *r* showed splitting as before, implying the CB[8] is kinetically trapped as seen for the previous systems.

Overall this allowed the models in Fig. 28 to be proposed, where it is most likely that the unbound naphthyl will be conformationally confined close to the external surface of CB[8] due to its hydrophobicity.

1.7.7 Np_2Vio-CB[7]

With CB[7], more complex spectra are observed as shown in Fig. 29. Previously upon slow introduction of CB[7] or CB[8] in steps, well defined peaks can be observed for the newly formed complex. This means a simple two

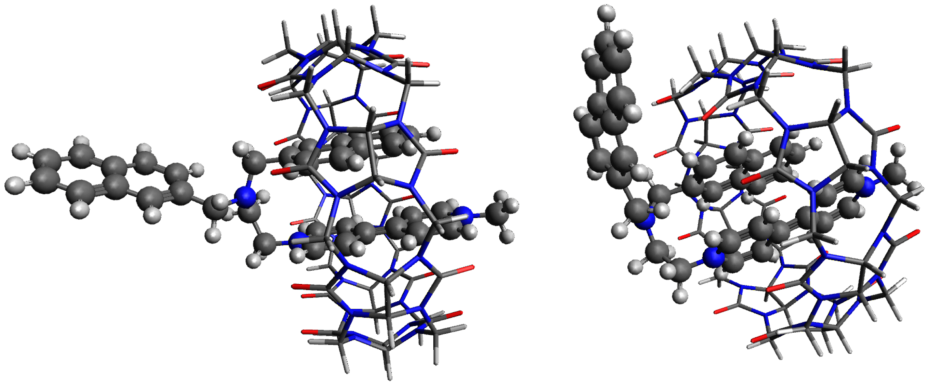

Figure 28: The proposed complexes of Np_2V with 1 CB[8] molecule forming an asymmetric complex. The position of CB[8] was derived from the ^{1}H NMR experiments and optimisation was carried out with MMFF94 molecular mechanics in Avogadro v1.2.

component mixture was present, unbound free guest molecules and guest-CB[n] conjugates (as in Fig. 2 in the main text with 0.8 *equiv.* CB[8]). In the case of CB[7] addition to Np_2V, the spectrum broadened substantially and lost much of its intensity. This implies that after introduction of a small amount of CB[7], some of the V can be bound and then the two free Np groups will stack, likely in an intermolecular fashion, into higher ordered structures. A suggested model is shown in Fig. 30.

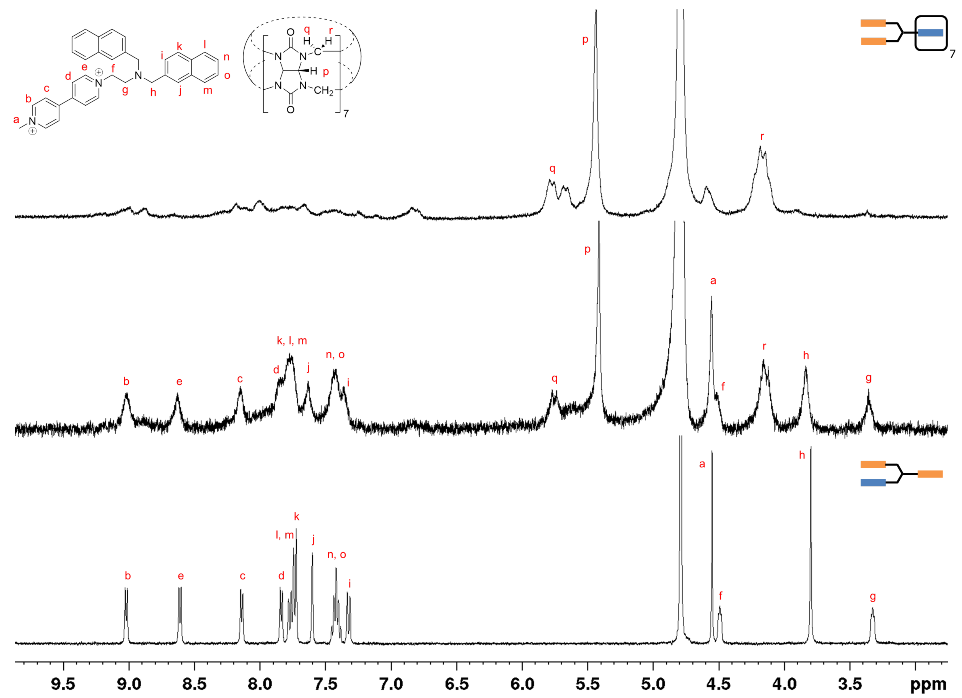

Figure 29: ^{1}H NMR spectrum of Np_2V in D_2O (bottom) with 0.2 (middle) and 1.0 *equiv.* (top) of CB[7].

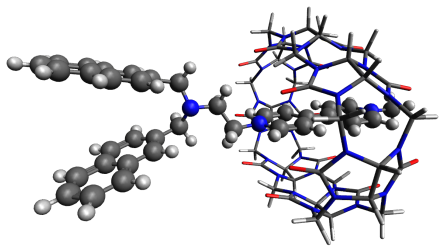

Figure 30: The proposed complex of Np_2V with 1 CB[7] molecule. Optimisation was carried out with MMFF94 molecular mechanics in Avogadro v1.2..

1.8 Photo-isomerisation of $AzoV_2$ Complexes

^{1}H NMR stacks following the photo-isomerisation of $AzoV_2$ and its complexes with CB[7] and CB[8] are shown in this section.

By UV-Vis, the $AzoV_2$ at a low concentration (19.2 μM) displays fast isomerisation kinetics. After 10 mins of UVA exposure, the molecule had reached its photo-stationary state, evidenced by no more change in the UV-Vis trace upon further exposure. As shown in Fig. 31, in response to UVA light the peak corresponding to the *E* isomer at 333 nm reduced ($\pi - \pi^*$), and the peak corresponding to the *Z* isomer ($n - \pi^*$) at 423 nm was enhanced.[7] The peak corresponding to V was also observed to blue shift from 260 nm to 257 nm upon the conformational change. This was likely a result of the disruption of aromatic donor-acceptor interactions between *E*-Azo and V, as the *Z* isomer will overlap less and be more polar. Upon heating, the isomerisation can be reversed to drive formation of predominately the *E* isomer, which will relax back to the initial equilibrium state after several hours.

In the presence of two CB[8] molecules, *i.e.* the 2:1 complex, this isomerisation is severely inhibited. Even after 1 h of UVA exposure, the isomerisation could not be driven further than observed in Fig. 31. Here, the peak of the *E* isomer at 327 nm (blue-shifted due to complexation and enhanced donor-acceptor effects) was observed to decrease in intensity only a small amount, with little observable change for the peak hidden at 423 nm. The V peak at 263 nm (red-shifted from complexation) was completely unchanged. This implied that the inclusion of the Azo group within the cavity altered the photo-isomerisation equilibrium due the *Z* isomer not being able to form a heteroternary complex. This was likely due to the high K_a and slow dissociation kinetics. However, upon heating to recover the *E* isomer a significant change was observed in the spectrum. The 327 nm peak was recovered well, but the 263 nm peak had loss some intensity, and there appeared to me more intensity from the peak at 423 nm. It was thought that a small population of the $AzoV_2 \cdot (CB[8])_2$ complex had altered its structure, likely being stabilised between the CB[8] molecules, however due the low population the exact structure could not be elucidated from NMR studies.

In contrast, in the presence of two CB[7] molecules the isomerisation proceeded as for uncomplexed $AzoV_2$. A large reduction of similar magnitude was observed in the peak at 332 nm (blue shifted 1 nm upon complexation), and the appearance of a peak at 424 nm occurred. Therefore, no inhibition of the isomerisation was observed. The V peak was also of great interest as an enhancement of signal was observed along with a large blue shift to

253 nm. In addition, the peak shape altered significantly. This implied that after isomerisation had occurred, some of the V was in a significantly altered environment, potentially even displaced from the CB[7] cavity by some binding of the Z isomer of Azo to CB[7] as has been observed in the literature with weaker binding constants ($K_a = 10^{3-4}$ M^{-1}), or by the more polar Z isomer gaining stability adjacent to CB[7].[8] The process did not appear to be completely reversible, however, not fully recovering the initial equilibrium state, likely due to the Z isomer binding to CB[7].

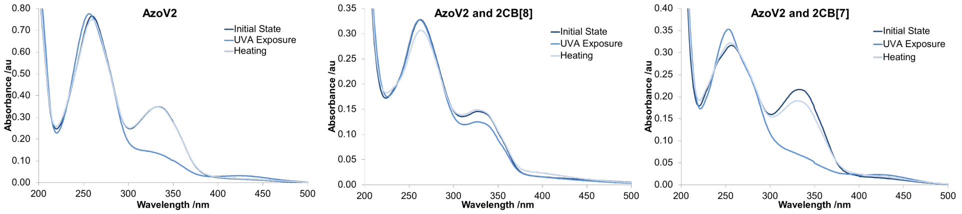

Figure 31: UV-Vis spectra demonstrating the photo-physical properties of AzoV$_2$, AzoV$_2$ with 2 *equiv.* CB[8], and AzoV$_2$ with 2 *equiv.* CB[7]. The initial photo-stationary state is shown, followed by UVA exposure until the spectrum remains unchanged, then heated at 70 ° C until the spectrum remains unchanged.

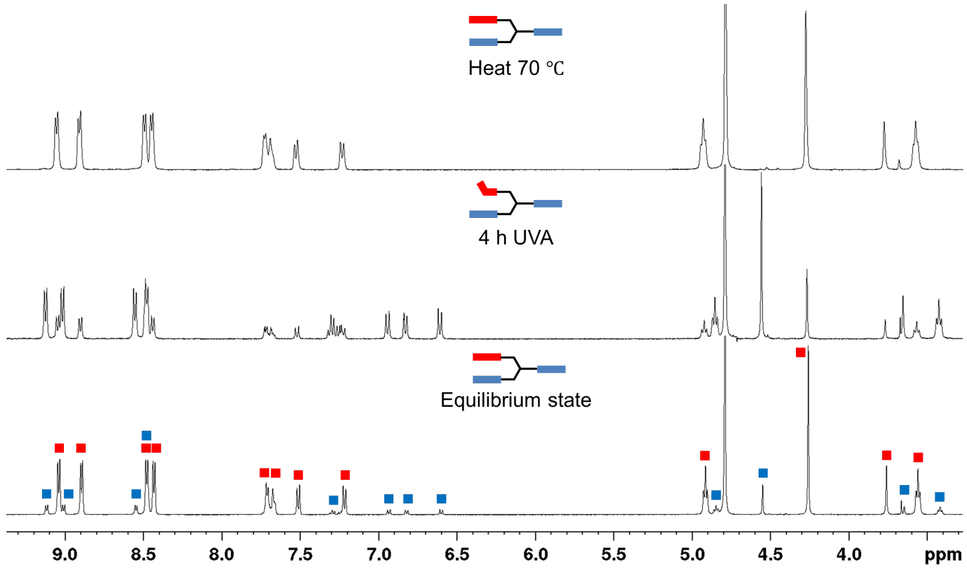

Figure 32: Stacked ^{1}H NMR spectra of **AzoV**$_2$ following its photo-isomerisation. From the bottom the equilibrium state is shown, then predominately the Z isomer after 4 h UVA irradiation, then predominately the E isomer after heating. Peaks labelled red correspond to the E isomer, and those in blue to the Z isomer.

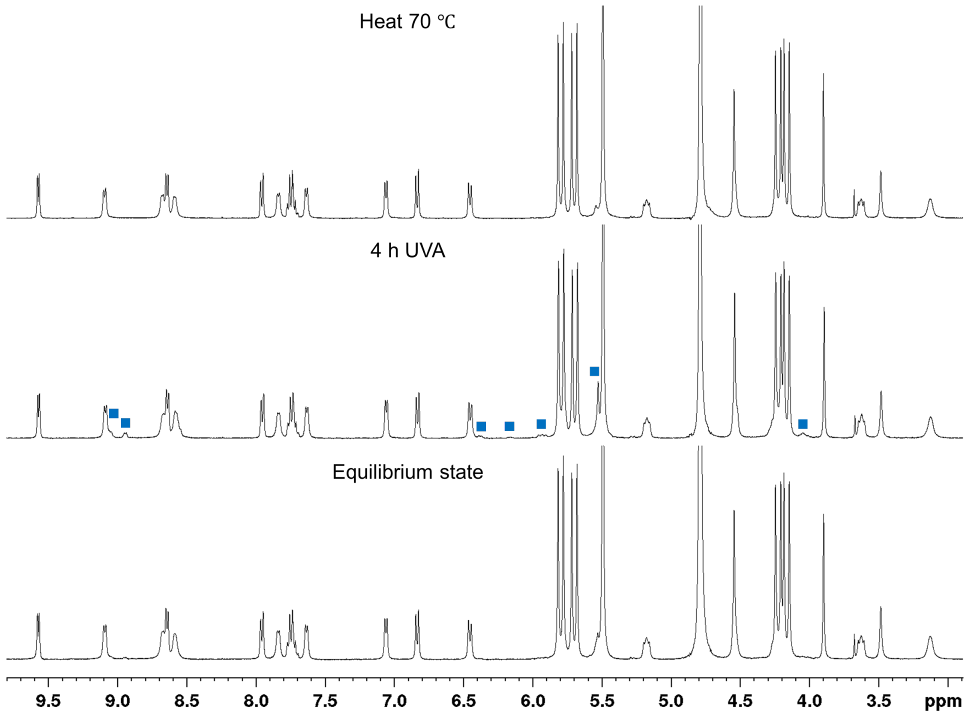

Figure 33: Stacked ^{1}H NMR spectra of the **AzoV$_2$·CB[8]** complex following its photo-isomerisation. From the bottom the equilibrium state is shown, then predominately the Z isomer after 4 h UVA irradiation, then predominately the E isomer after heating. Peaks labelled blue correspond to the Z isomer.

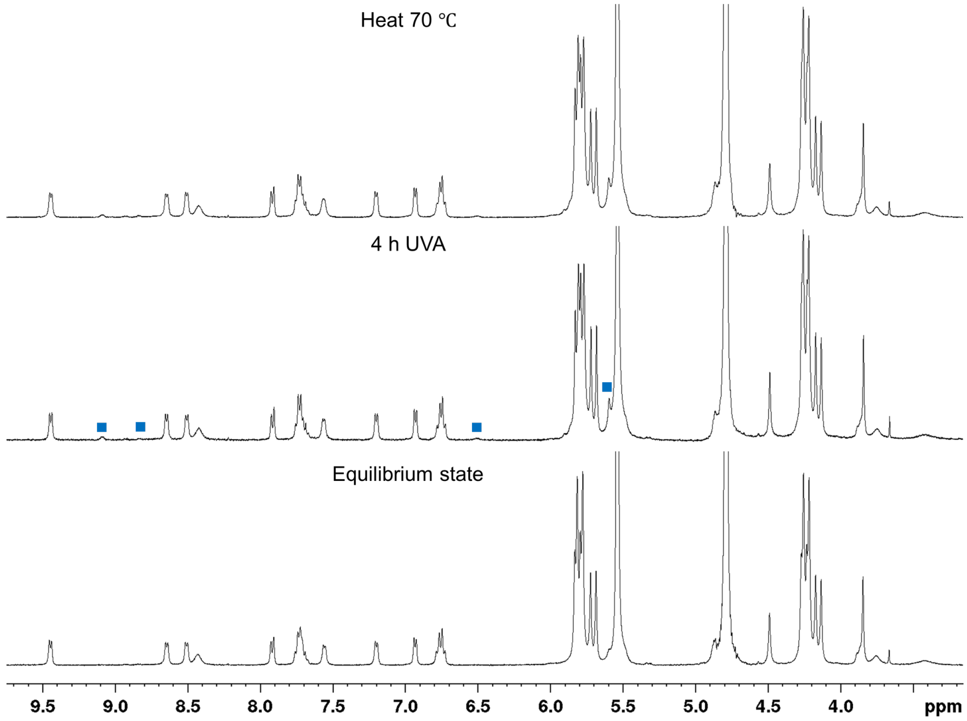

Figure 34: Stacked ^{1}H NMR spectra of the $\mathbf{AzoV}_2\cdot(\mathbf{CB[8]})_2$ complex following its photo-isomerisation. From the bottom the equilibrium state is shown, then predominately the Z isomer after 4 h UVA irradiation, then predominately the E isomer after heating. Peaks labelled red correspond to the E isomer, and those in blue to the Z isomer.

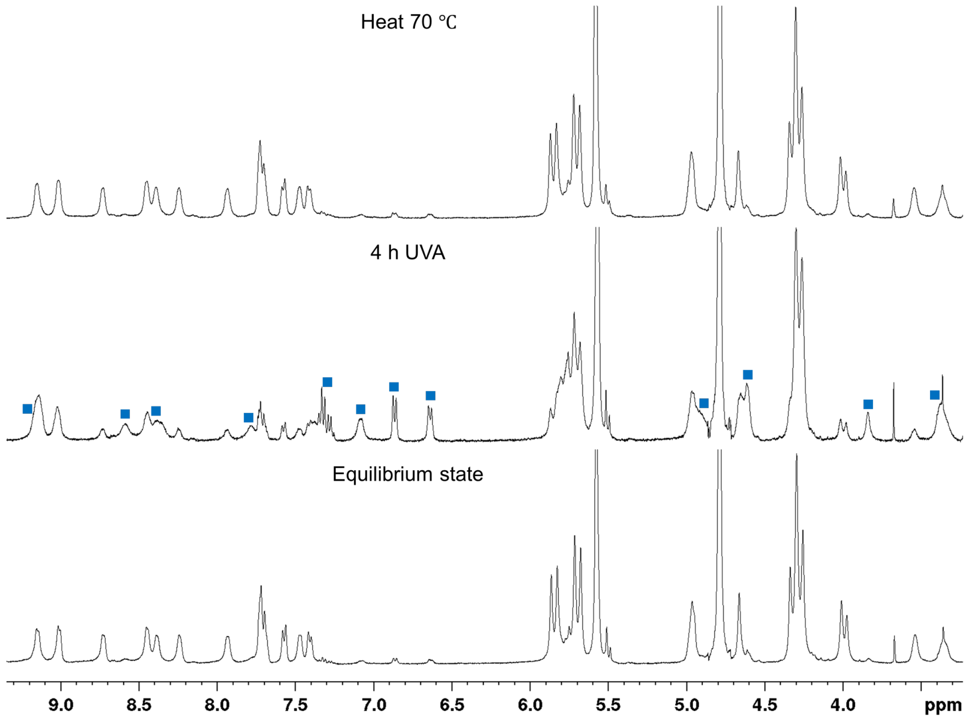

Figure 35: Stacked ^{1}H NMR spectra of the **AzoV**$_2$·**CB[7]** complex following its photo-isomerisation. From the bottom the equilibrium state is shown, then predominately the Z isomer after 4 h UVA irradiation, then predominately the E isomer after heating. Peaks labelled red correspond to the E isomer, and those in blue to the Z isomer.

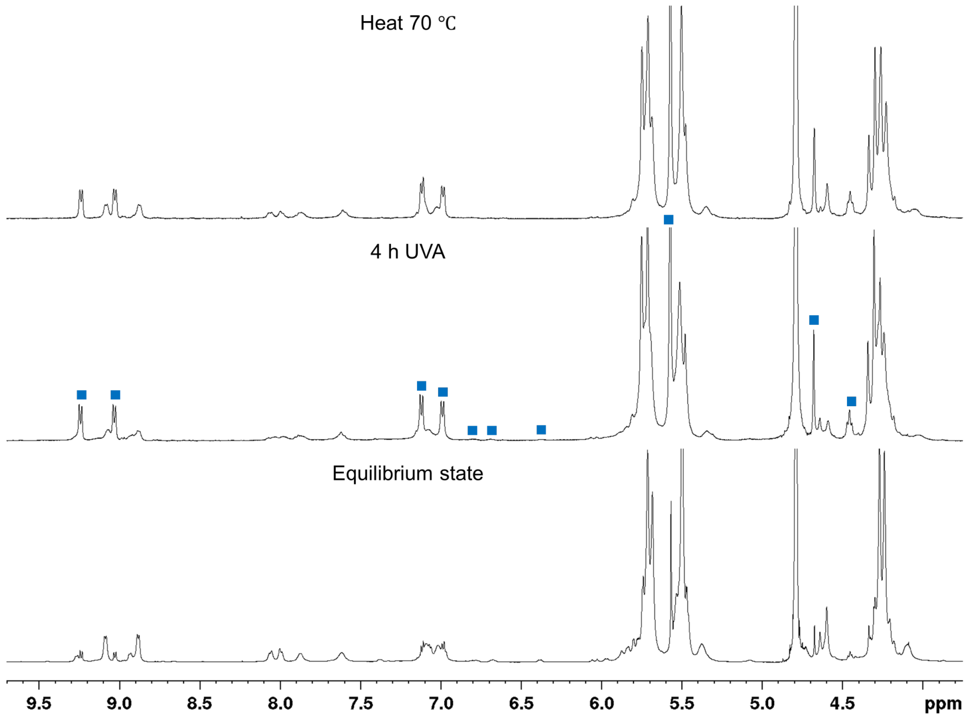

Figure 36: Stacked ^{1}H NMR spectra of the **AzoV**$_2$·**(CB[7])**$_2$ complex following its photo-isomerisation. From the bottom the equilibrium state is shown, then predominately the *Z* isomer after 4 h UVA irradiation, then predominately the *E* isomer after heating. Peaks labelled red correspond to the *E* isomer, and those in blue to the *Z* isomer.

1.9 Further Complexation with NpV$_2$-2CB[8]

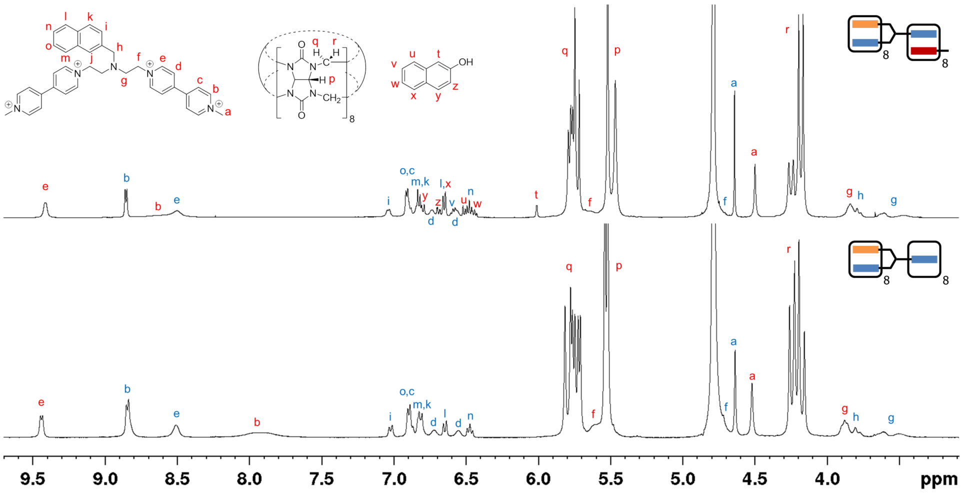

Figure 37: Stacked ^{1}H NMR spectra of NpV$_2$·(CB[8])$_2$ in D$_2$O (bottom spectrum), followed by addition of 1 *equiv.* of 2-naphthol (top spectrum). The peak assignments in red show proton environments outside of the CB[8] cavity or binding with 2-naphthol, and those in blue are inside the cavity as an intramolecular heteroternary 1:1:1 complex.

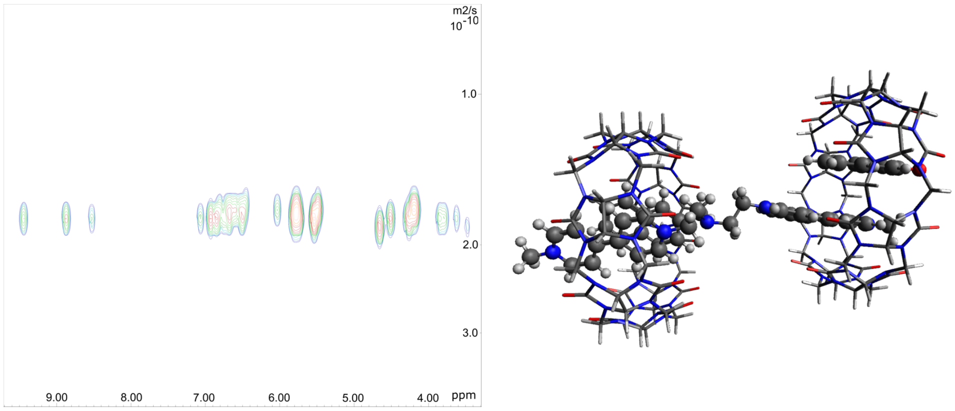

Figure 38: The DOSY NMR spectrum for the NpV$_2$·(CB[8])$_2$ complex with 2-naphthol (left), and the calculated model carried out with MMFF94 molecular mechanics in Avogadro v1.2. (right).

After formation of the NpV$_2$-2CB[8] complex, it was expected that the

binary complex between V and CB[8] could undergo further binding to a suitable guest molecule to selectively give another heteroternary complex in a self-sorting manner. 2-naphthol was selected as a suitable second guest as it is similar to the already present heteroternary complex, with a reported binding constant of $K_a = 6.2 \times 10^5$ M^{-1} to the V-CB[8] complex.[5] After introduction of solid 2-naphthol, the expected complex was formed resulting in a significant colour change from orange to deep red. The corresponding ^{1}H NMR spectrum stack shown in Fig. 37 shows significant differences to evidence complexation. The peaks labelled in blue corresponding to the V-Np intramolecular complex remain almost completely unchanged upon addition of 2-naphthol to $NpV_2{\cdot}(CB[8])_2$. The peaks labelled in red corresponding to the binary V-CB[8] complex display many changes as the 2-naphthol is encapsulated forming an intermolecular heteroternary complex. The peaks corresponding to 2-naphthol (t - z) are upfield shifted and overlap with the existing naphthyl peaks; this shows that they are also encapsulated within CB[8] as a heteroternary complex. The V peak b showed a significant downfield shift of 0.6 ppm, becoming more similar to the intramolecular peak b. Overall the CB[8] was concluded to fully encapsulate the 2-naphthol, and would be centred over protons c and d. Shifts were also observed for the CB[8] peaks, with p - r shifting upfield for the CB[8] complexing with 2-naphthol, likely a result of the added π ring current within the cavity.

DOSY NMR (Fig. 38) showed a single species was present with $D = 1.78 \times 10^{-10}$ m^2 s^{-1} and diameter = 2.76 nm, in line with the previously discussed DOSY results. A model is also shown in Fig. 38, allowing visualisation of the complex.

1.10 Mixtures with ADA·HCl

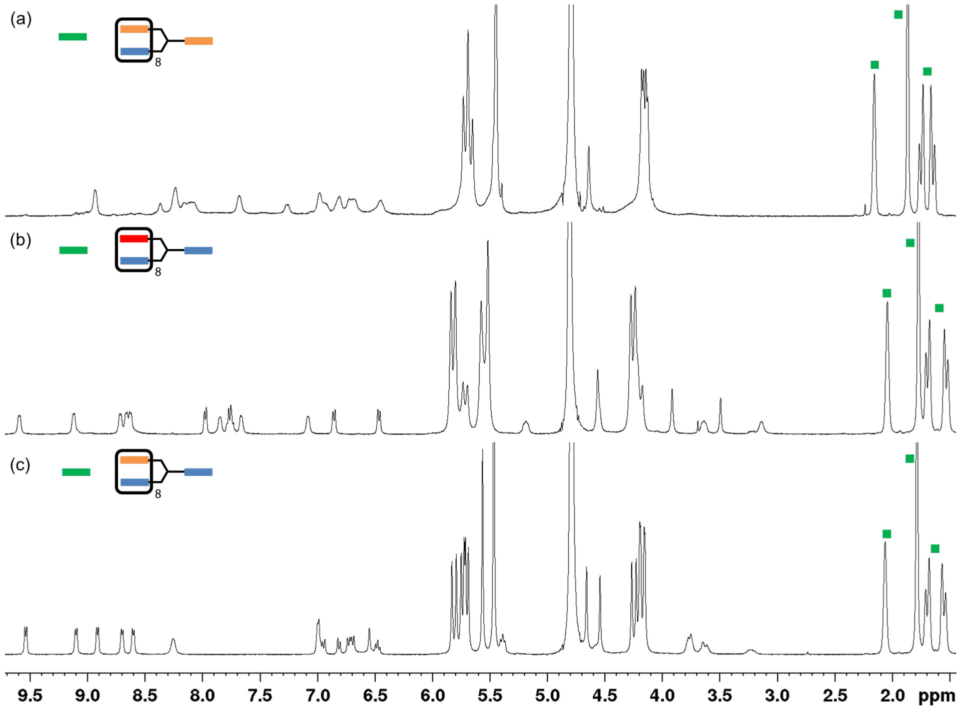

Figure 39: Stacked ^{1}H NMR spectra of the addition of excess ADA·HCl to (a) $Np_2V{\cdot}CB[8]$, (b) $AzoV_2{\cdot}(CB[8])_2$, and (c) $NpV_2{\cdot}(CB[8])_2$. ADA·HCl could only displace the binary complexes with CB[8] and not the intramolecular heteroternary complexes. Schematics of the complexes present have been included for each step and peaks labelled with green squares correspond to ADA·HCl.

The chemical responsiveness and self-sorting nature of these complexes was also explored through simple introduction of ADA·HCl, which has a binding constant reported to be 4.2×10^{12} M^{-1} for CB[7] and measured in this work as 3.1×10^9 M^{-1} for CB[8].[5,9] The ^{1}H NMR was followed upon titration of this chemical stimulus into a solution of the complexes, and *vice versa*.

For the $NpV_2{\cdot}(CB[8])_2$ and $AzoV_2{\cdot}(CB[8])_2$ complexes as shown in Fig. 39 1 CB[8] can be displaced from the complex, being the weaker binary complex between V and CB[8]. The intramolecular complex displayed stronger binding as previously discussed, where due to its much higher K_a it could not

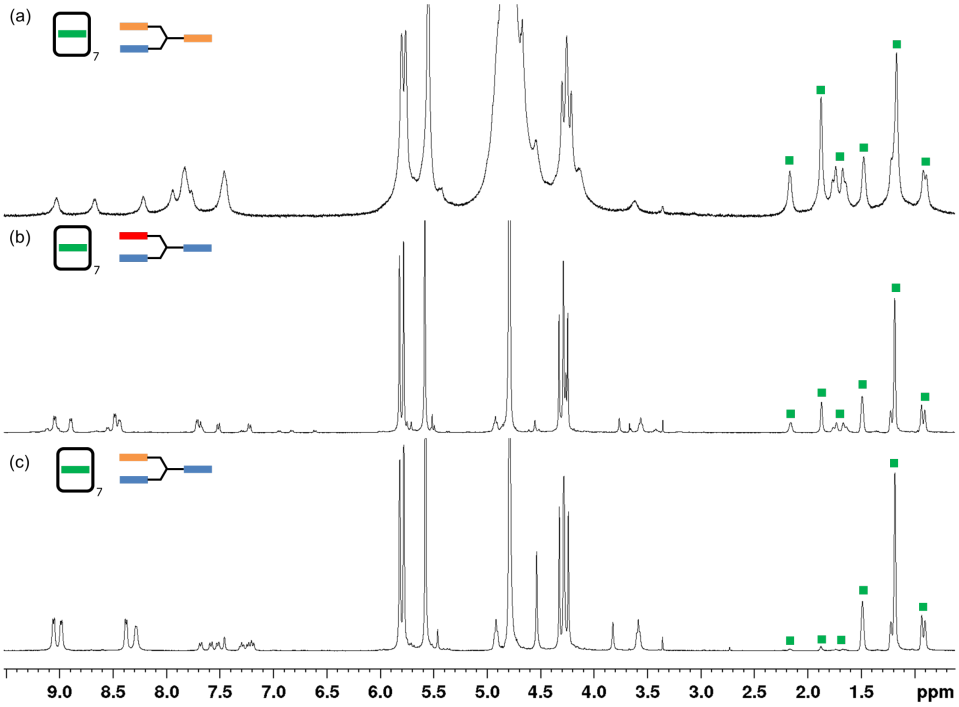

Figure 40: Stacked ^{1}H NMR spectra of the addition of excess ADA·HCl to (a) $Np_2V\cdot(CB[7])_2$, (b) $AzoV_2\cdot(CB[7])_2$, and (c) $NpV_2\cdot(CB[7])_2$. In each case the initial $AB_2\cdot(CB[7])_2$ complex was displaced resulting in an ADA·HCl·CB[7] complex and the unbound AB_2 molecule. Schematics of the complexes present have been included for each step and peaks labelled with green squares correspond to ADA·HCl.

be displaced after heating to 70 °C for 24 h in 3 times excess ADA·HCl. The same behaviour was observed for Np_2V where no change was observed upon addition of ADA·HCl. This provided the opportunity to control supramolecular topology in complex aqueous mixtures. In contrast, the CB[7] complexes could be completely disassembled in response to ADA·HCl addition as shown in Fig. 40. This is because of the much stronger binding of ADA·HCl to CB[7] than that of the AB_2 molecules, resulting in irreversible displacement.

The self-sorting nature of this system was further investigated by inverting the addition of components. A 1:1 complex of ADA·HCl with CB[8] was pre-assembled, to which different guests were added. The stacked spectra for each separate experiment are shown in Fig. 41. In each case it

was clear that the addition of the guest molecules completely displaced the ADA·HCl·CB[8] complex, however only 1 CB[8] is associated with each guest forming the strong intramolecular complex. Further addition of guest results in a controlled mixture of AB_2·CB[8], AB_2, and ADA·HCl. Therefore, in the presence of competing guest, the mixture will self-sort to form exclusively the intramolecular complexes, with no evidence of the remaining V binding with CB[8]. This data supports the ITC measurements undertaken, where ADA·HCl was used as a competitive guest in order to calculate the binding constant for the intramolecular complexation.

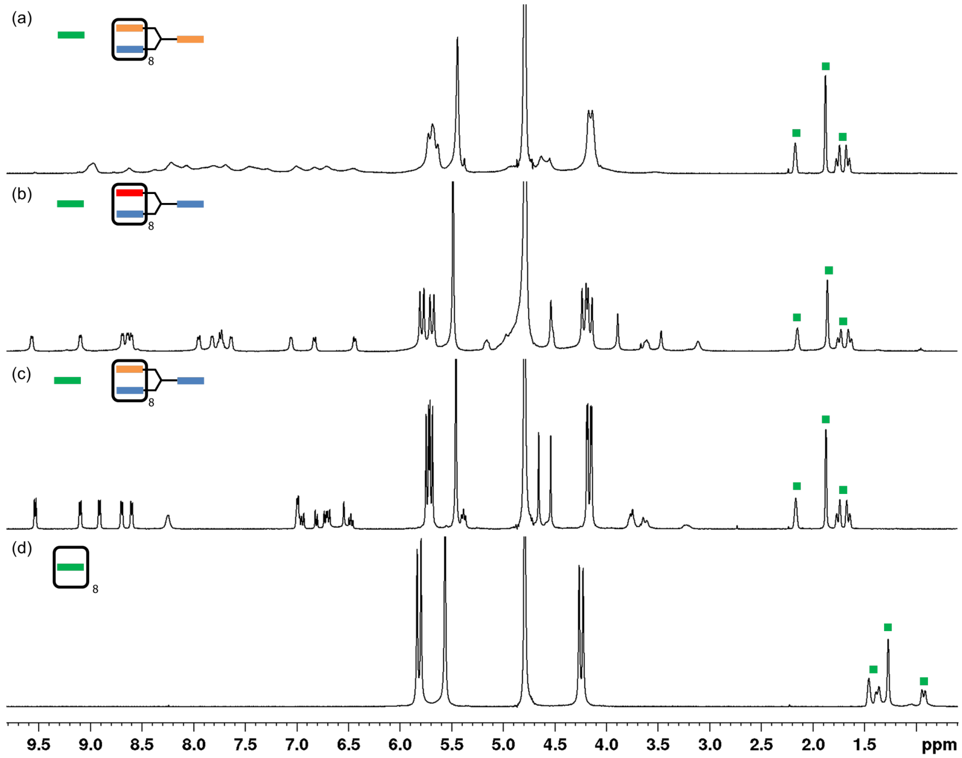

Figure 41: Stacked ^{1}H NMR spectra of the addition of Np_2V (a), $AzoV_2$ (b), NpV_2 (c) to the binary complex of ADA·HCl with CB[8] (d) in D_2O. Schematics of the complexes present have been included for each step and peaks labelled with green squares correspond to ADA·HCl.

1.11 UV-Vis Spectroscopy

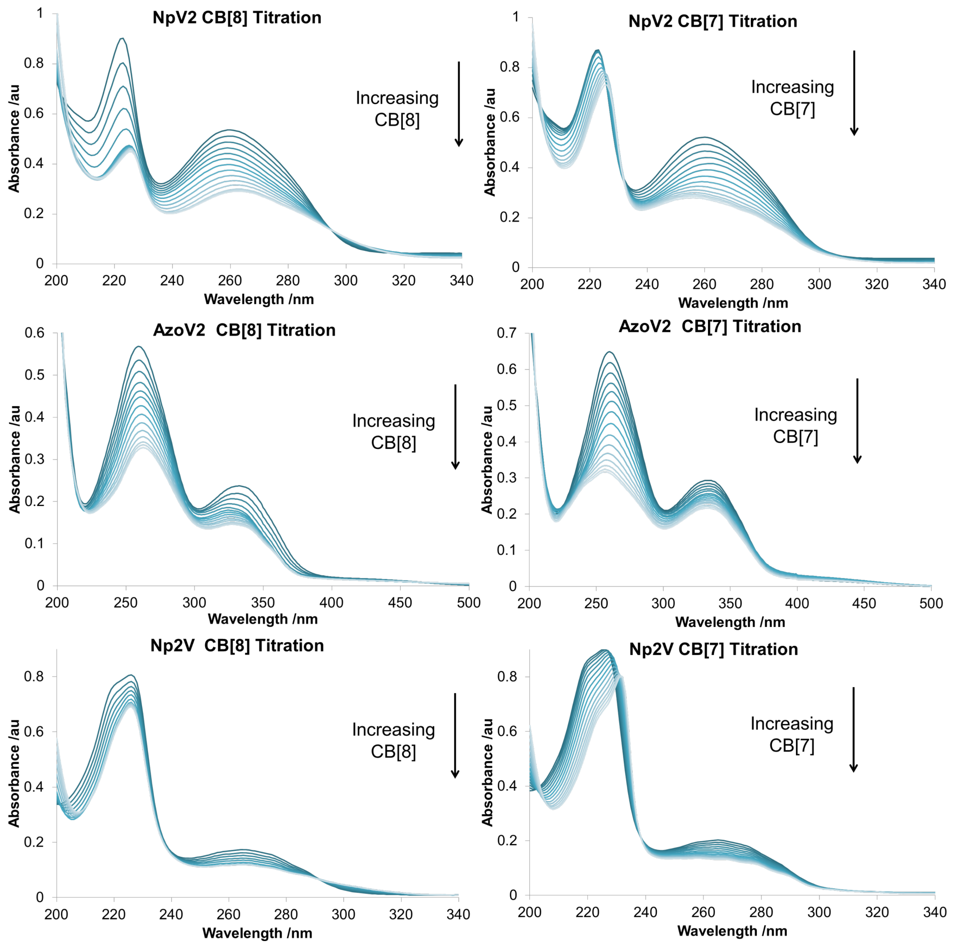

Figure 42: UV-Vis spectra of each AB_2 molecule followed by titration with CB[8] or CB[7].

UV-Vis spectrophotometry is a useful technique for monitoring CB[n] host-guest binding as this typically induces many changes in the electronic properties of the guests. Dilute solutions of each AB_2 molecule were titrated with CB[7] or CB[8] solutions, and their spectra are shown in Fig. 42.

The initial NpV_2 spectrum has λ_{max} at 223 and 260 nm. Naphthyl groups typically have an intense absorption band around 220 nm and a minor band around 280 nm, and here there is some evidence of a shoulder peak

being present with NpV_2 at *c.a.* 285 nm. V has an intense absorption at 260 nm overlapping with this.[7] Upon addition of CB[8], the intramolecular heteroternary complex will first be formed up to 1 *equiv.*, followed by the binary complex up to 2 *equiv.*. This is reflected in the significant drop in intensity for the naphthyl peak at 223 nm upon complexation followed by no more significant changes. The viologen peak at 260 nm also decreases in intensity. As the viologen peak reduced in intensity upon both being encapsulated within CB[8], the secondary band for naphthyl became more clear, with an isobestic point observed at 295 nm. With introduction of CB[7], the naphthyl peak does not initially change until 1 *equiv.* of CB[7] has been added, whereupon a red shift of the band to 226 nm was observed with a small reduction in intensity. This was thought to be due to confinement of the naphthyl between the electronegative carbonyl portals of the two CB[7] molecules (see Fig. 20). The viologen peak reduces in intensity as binary complexation occurs.

$AzoV_2$ showed λ_{max} of 260 and 332 nm. The peak at 260 nm was from viologen, similar to NpV_2. The peak at 333 nm corresponded to the $\pi - \pi^*$ transition of E-azobenzene.[7] Upon introduction of 1 and 2 *equiv.* of CB[8], the viologen peak decreased in intensity. The azobenzene peak decreased rapidly in intensity up to 1 *equiv.* of CB[8] coinciding with intramolecular complexation. With CB[7], the viologen peak decreased in intensity and the azobenzene peak showed a small decrease in intensity but was otherwise unaltered.

Np_2V showed λ_{max} of 226 and 265 nm, corresponding to the naphthyl and viologen groups respectively. There is also a shoulder peak present at 220 nm, likely due to the existence of the two naphthyl groups in different conformations or through intramolecular stacking between them. Upon introduction of 1 *equiv.* of CB[8], the intensity of the naphthyl peak at 226 nm decreases and the shoulder peak is mostly lost as stacking interactions disappear. The viologen peak decreases in intensity in a similar fashion, with an isobestic point observed at 291 nm due to the secondary naphthyl band as for NpV_2. With CB[7], as the viologen is encapsulated with up to 1 *equiv.* it decreases in intensity. As evidenced by NMR experiments, introduction of CB[7] results in stacking of naphthyl components as the viologen becomes encapsulated. The main naphthyl peak red shifts to 232 nm, and the shoulder peak becomes much clearer, also red shifting to 224 nm. This corresponds to enhanced stacking between the aromatic naphthyl units.

1.12 ITC

Molecule	$K_{1CB[7]}$	$\Delta H_{1CB[7]}$	$T\Delta S_{1CB[7]}$	$K_{2CB[7]}$	$\Delta H_{2CB[7]}$	$T\Delta S_{2CB[7]}$
NpV_2[b]	1.5×10^6	-14.2	22.2	-	-	-
$AzoV_2$	4.8×10^7	-28.2	15.6	2.3×10^5	-13.0	17.6
Np_2V[b]	2.1×10^6	-64.7	-28.7	-	-	-

[a] K_a was in units of M^{-1}, ΔH and $T\Delta S$ in units of kJ mol^{-1}.
[b] Poor fitting was observed due to overlapping thermodynamic processes.

Table 1: Thermodynamic data for the complexes with CB[7].

ITC isotherms and their fitting curves with different models are shown in this section. Due to the limited solubility of neat CB[8],[5] the guest molecules NpV_2, $AzoV_2$ and Np_2V (1 mM) with multiple potential guest binding sites were titrated into a calorimetric cell containing CB[7] (61 μM) or CB[8] (51 μM). However, this lead to complex isotherms as shown in Fig. 44, as initially the guest molecule will be in the presence of excess CB[n]. NpV_2 and $AzoV_2$ will therefore bind with two CB[n]s to form intramolecular heteroternary complexes (NpV_2-2CB[8] and $AzoV_2$-2CB[8]) respectively, resulting in a binding stoichiometry of 0.5. Further addition of the guest molecule resulted in the formation of binary complexes with CB[7] or molecular hinges with CB[8]. This behaviour is summarised in Fig. 43. Attempted fitting of a multiple binding sites model to the first step of this isotherm gave results showing the first binding for CB[8] $K_{1CB[8]} > 10^{10}$ M^{-1}, and for CB[7] the overall binding for both viologen guests was $K_a > 10^9$ M^{-2}. With Np_2V binding to CB[8], an equally high K_a was observed, and competing stacking interactions prevented any meaningful fit with CB[7].

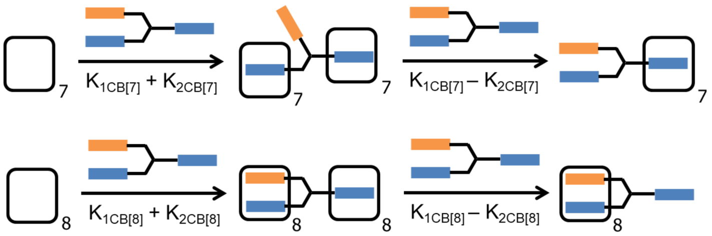

Figure 43: Schematic diagram showing the binding events observed in the ITC measurements shown in Fig. 44 for NpV_2 and $AzoV_2$.

As the strength of binding was often higher than that observable with ITC (10^{2-7} M^{-1}), a displacement titration was employed following literature

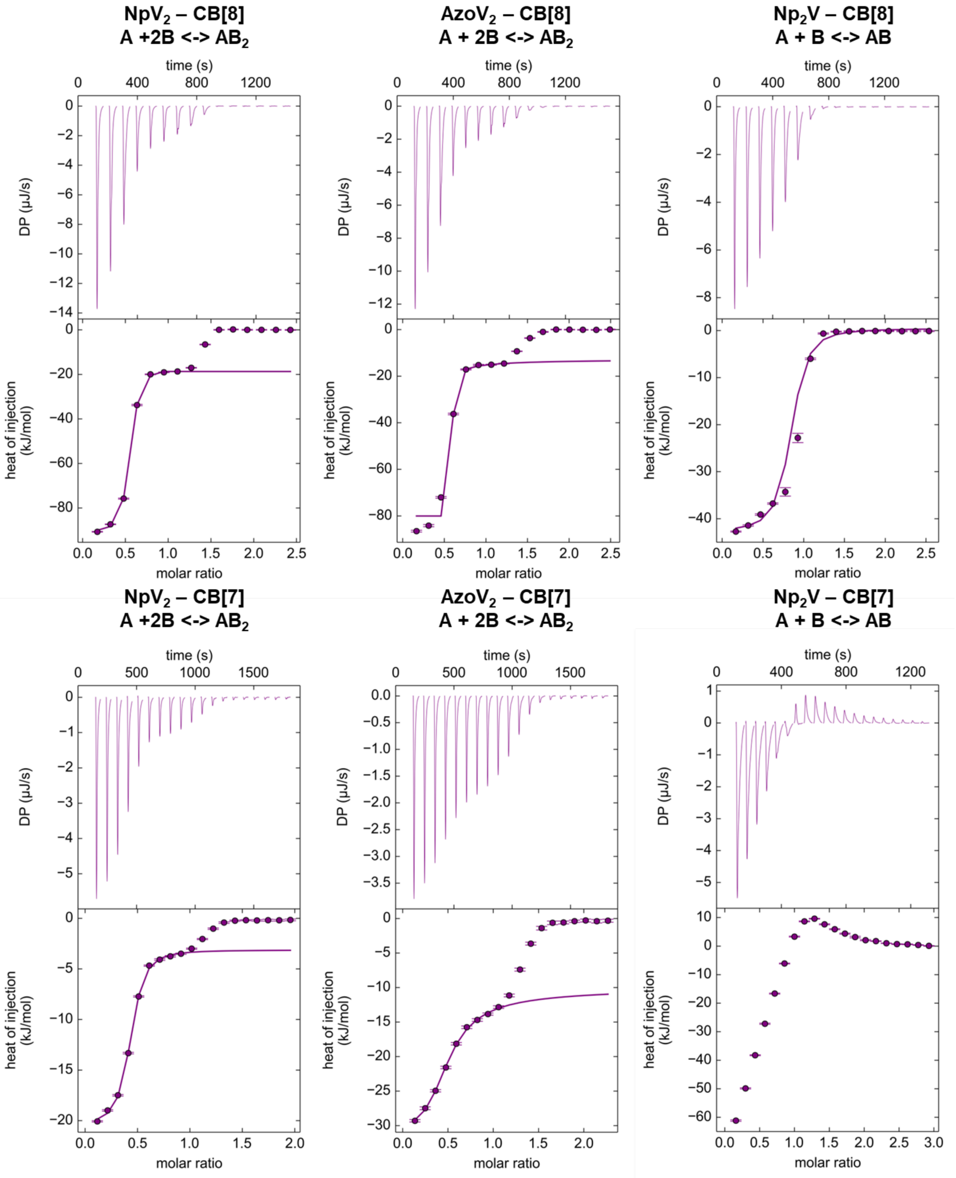

Figure 44: ITC isotherms for cells containing CB[7] (61 μM) or CB[8] (51 μM) titrated with each AB_2 (1 mM) molecule in H_2O at 298.15 K.

procedures.[10–12] In a typical experiment, the calorimetric cell contains both CB[8] (30 μM) and a weaker binding competitive guest (0.85 mM). This is

titrated with the guest molecule (0.4 mM), and the K_a and ΔH can be calculated as in Eq. 1, where K_{exp} is the experimentally measured K_a, $[Comp]$ is the concentration of the competitive guest, and K_{Comp} is the K_a for the weaker binding guest to CB[n]. As characterisation of ADA·HCl binding with CB[8] has not yet been reported with ITC, an initial displacement titration was carried out with methyl viologen as a competing guest as in Fig. 45, with results shown in the main text in Table 2.

$$K_a = K_{exp}(1 + [Comp] \times K_{Comp})$$
$$\Delta H = \Delta H_{exp} + \Delta H_{Comp}\frac{K_{Comp}[Comp]}{1 + K_{Comp}[Comp]} \quad (1)$$

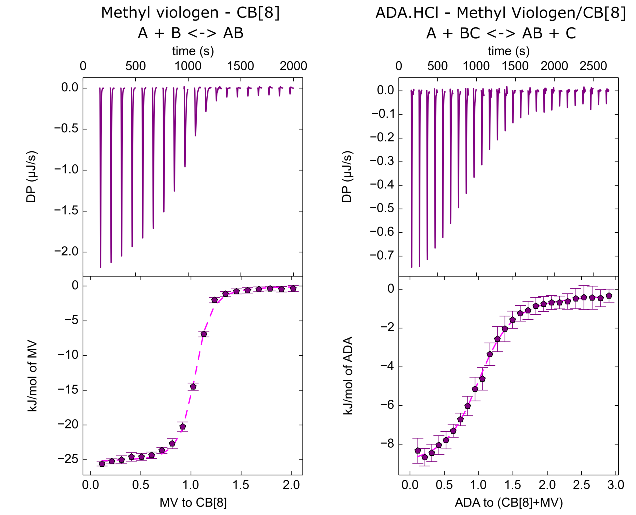

Figure 45: ITC titrations of methyl viologen into CB[8], and a competitive binding titration of ADA.HCl into methyl viologen-CB[8] at 298.15 K.

With this information in hand, displacement titrations with ADA·HCl (0.4 mM) as a competitive guest in the cell with CB[8] (30 μM) and NpV_2, $AzoV_2$ or Np_2V (0.3 mM) in the syringe were carried out as shown in Fig. 46. The binary complex was shown not to form in the presence of ADA·HCl, allowing a simple hetero-association model for fitting of the isotherm to be used. Results are shown in the main text in Table 2, and here in the ESI in Table 1.

With CB[7], binary complexation of the first and second CB[7] were expected to be similar and so a displacement titration could not be carried

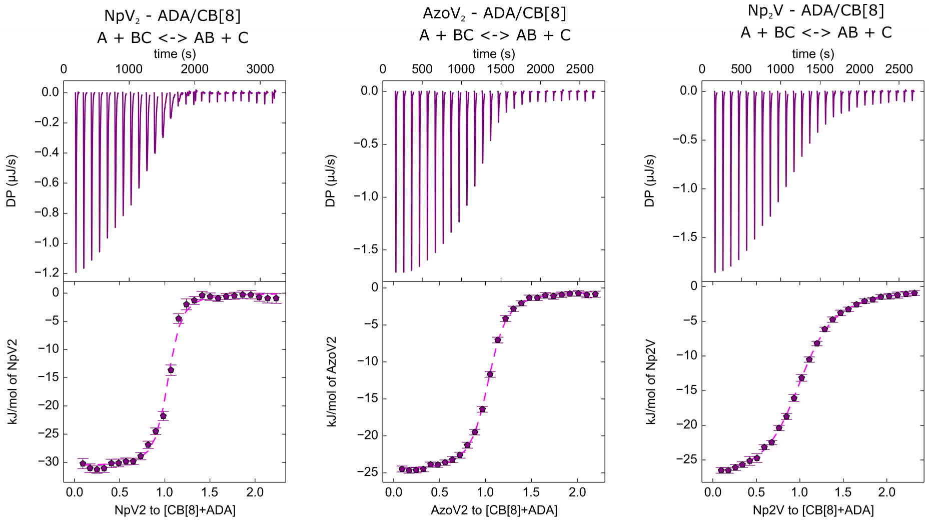

Figure 46: ITC isotherms for cells containing CB[8] (30 μM) and ADA.HCl (0.4 mM) titrated with each guest (0.3 mM) molecule at 298.15 K.

out. However, CB[7] is much more water-soluble than CB[8] [5] and so the titration could be inverted and the complexation events separated as in Fig. 47. The cell contained NpV_2, $AzoV_2$ or Np_2V (51 μM), and the syringe contained CB[7] (0.69 mM). With a multiple binding sites model, reasonable fits could be carried out for $AzoV_2$ with thermodynamic results summarised in the main text. The NpV_2 result however could not be reliably fit due to overlap between the first and second binding steps. If a hetero-association model is used, the first step can be fit for the binding of 1 CB[7]. Np_2V and CB[7] again had competing stacking interactions interfering with the fit, leading to unreliable results.

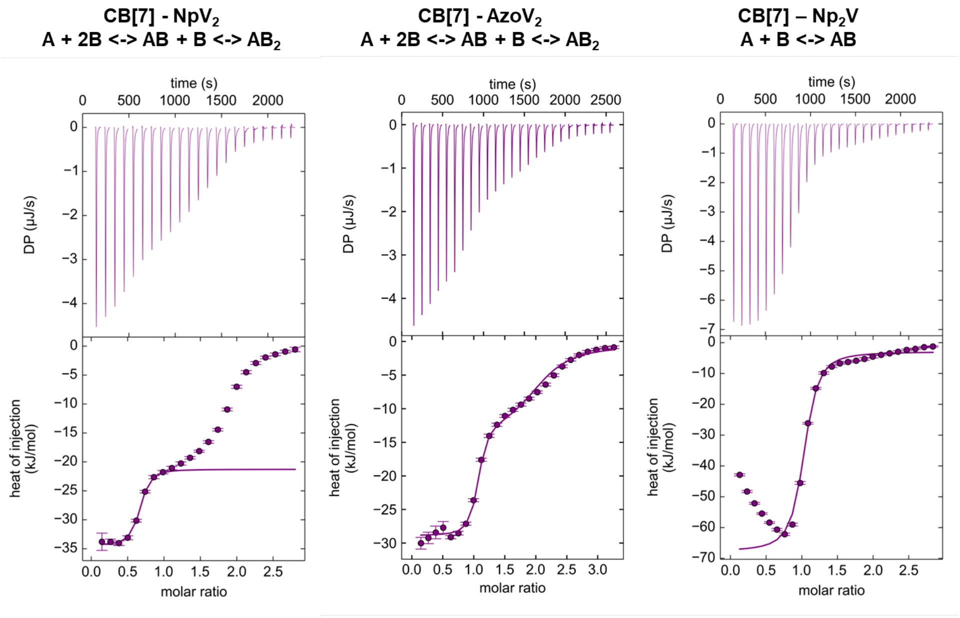

Figure 47: ITC isotherms for cells containing AB_2 or A_2B (51 μM) titrated with CB[7] (0.69 mM) in H_2O at 298.15 K.

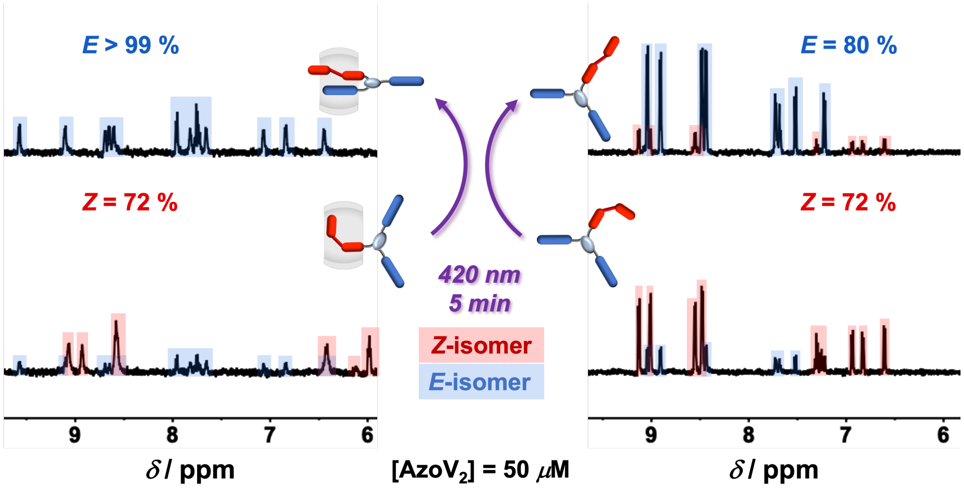

Figure 48: ^{1}H NMR spectra of Z to E isomerization of $AzoV_2$ (50 μM) in the presence (left) and absence (right) of CB[8] by 420 nm light for 5 min.

References

[1] C. A. Brautigam, H. Zhao, C. Vargas, S. Keller, P. Schuck, *Nat. Protoc.* **2016**, *11*, 882–894.

[2] S. Keller, C. Vargas, H. Zhao, G. Piszczek, C. A. Brautigam, P. Schuck, *Anal. Chem.* **2012**, *84*, 5066–5073.

[3] F. Tian, N. Cheng, N. Nouvel, J. Geng, O. A. Scherman, *Langmuir* **2010**, *26*, 5323–5328.

[4] P. Stawski, M. Sumser, D. Trauner, *Angew. Chemie - Int. Ed.* **2012**, *51*, 5748–5751.

[5] S. J. Barrow, S. Kasera, M. J. Rowland, J. Del Barrio, O. A. Scherman, *Chem. Rev.* **2015**, *115*, 12320–12406.

[6] L. Briquet, D. P. Vercauteren, E. A. Perpète, D. Jacquemin, *Chem. Phys. Lett.* **2006**, *417*, 190–195.

[7] F. Tian, D. Jiao, F. Biedermann, O. A. Scherman, *Nat. Commun.* **2012**, *3*, 1207.

[8] J. Wu, L. Isaacs, *Chem. - A Eur. J.* **2009**, *15*, 11675–11680.

[9] D. Sigwalt, M. Šekutor, L. Cao, P. Y. Zavalij, J. Hostaš, H. Ajani, P. Hobza, K. Mlinarić-Majerski, R. Glaser, L. Isaacs, *J. Am. Chem. Soc.* **2017**, *139*, 3249–3258.

[10] A. Velazquez-Campoy, E. Freire, *Nat. Protoc.* **2006**, *1*, 186–191.

[11] M. V. Rekharsky, T. Mori, C. Yang, Y. H. Ko, N. Selvapalam, H. Kim, D. Sobransingh, A. E. Kaifer, S. Liu, L. Isaacs, W. Chen, S. Moghaddam, M. K. Gilson, K. Kim, Y. Inoue, *Proc. Natl. Acad. Sci. USA* **2007**, *104*, 20737–20742.

[12] S. Moghaddam, C. Yang, M. Rekharsky, Y. H. Ko, K. Kim, Y. Inoue, M. K. Gilson, *J. Am. Chem. Soc.* **2011**, *133*, 3570–3581.
